# Spatio-temporal evolution of mortality in Cape Verde: 1995–2018

**DOI:** 10.1371/journal.pgph.0000753

**Published:** 2023-03-10

**Authors:** Domingos Veiga Varela, Maria do Rosário Oliveira Martins, António Furtado, Maria da Luz Lima Mendonça, Ngibo Mubeta Fernandes, Ivone Santos, Edna Duarte Lopes

**Affiliations:** 1 Department of Health Surveillance, National Institute of Public Health, Praia, Santiago, Cape Verde; 2 Institute of Hygiene and Tropical Medicine, Global Health and Tropical Medicine, New University of Lisbon, Lisbon, Portugal; 3 Pedro Gomes High School, Ministry of Education, Praia, Santiago, Cape Verde; 4 National Institute of Public Health, Health Research, Praia, Santiago, Cape Verde; 5 Integrated Surveillance and Response Service, National Directorate of Health, Praia, Santiago, Cape; University of Embu, KENYA

## Abstract

Located in West Africa, Cabo Verde is a low income country, with significant gains in health indicators. Mortality is an important demographic factor. Its analysis provides essential statistical data for the design, implementation and evaluation of public health programs. The propose of this work is to analyze the spatio-temporal evolution of mortality in Cabo Verde between 1995 to 2018. This is an observational, quantitative study that performs demographic analysis of mortality data from the Ministry of Health of Cabo Verde. Specific mortality rates from standardized causes were calculated considering the population of the country as a reference in the year 2010 and also the standardized rate for all causes on each island, aiming at comparing the islands. During the period under analysis, the number of deaths in men was always higher than that of women. The main causes of death were diseases of the circulatory system and with a higher incidence in women. São Nicolau, Brava, and Santo Antão islands have mortality rates, higher than the national level (2010–2018). The main cause of premature death in women as identified as diseases of the circulatory system, while in men it is injuries, trauma, poisoning and external causes. There was a 72% decrease in the mortality rate due to unclassified symptoms and clinical signs, and an increase in respiratory diseases and tumours. With the exception of diseases of the circulatory system, mortality rates in men are higher than in women for all the considered causes. A decrease in specific mortality rates by age group is expected for both sexes, with a greater gain in men in the younger age groups. With these data, it is intended to alert health decision-makers about the best strategies to be defined in the reduction of mortality in the country.

## Introduction

It is globally accepted that the average life expectancy of men is lower than that of women [1], with this ratio varying depending on the age group, despite a downward trend [2]. Worldwide data show that the mortality rate for men was approximately three times higher than for women in 2011 [3]. Some studies have shown that women have a longer life expectancy than men, with this difference varying between 4 and 5 years in developed countries [4,5].

The disparity in health status between the sexes depends on epidemiological, socioeconomic (social circumstances, poverty, investments in health structures) and behavioural (tobacco consumption, excessive alcohol use) factors and vary over time and space [1,4,6].

The studies by Cullen, et. al. (2015) [5] and Wong, et. al. (2017) [7], suggest that tobacco use and excessive alcohol consumption may be associated with a high incidence rate of lung cancer and liver cirrhosis in men. Although, results obtained by Wong et al. (2017) [7], have shown a decrease in the rates of men and an increase in those of women for most of the (developed) countries analysed.

Located 500 km off the west coast of Africa, Cabo Verde is an archipelago country, consisting of 10 islands, nine of which are inhabited. The country has a resident population of about 483,628 inhabitants (2021 Census). Only 10% of its territory is classified as arable land, and the country has limited mineral resources. The country economy is guided by tourism (which accounts for approximately 20% of GDP) with a temperate climate throughout the year. The territory fragmentation creates significant connectivity challenges as well as obstacles to the delivery or provision of services, including energy, water, education and healthcare. Despite the challenges associated with the small island economy, Cabo Verde has seen remarkable economic progress since 1990, largely driven by the rapid development of tourism, particularly of all-inclusive resorts, as well as considerable social developments resulting from the implementation of strong social policies. since the 70s. In Cabo Verde, there was a high mortality rate in the 15 to 49 age group, being about two times higher in men when compared to women, in 2016, and three (03) times higher in 2017 [8]. A study developed by Delgado (2013) [9] is the only in the country portraying mortality in the population. However, the data date back to 2010, in addition to being limited to the São Vicente, with data for the year 2010.

Thus, it is necessary to study the evolutionary pattern of mortality distribution in Cabo Verde over the years, to analyse the reasons for the high burden of premature mortality and the discrepancy between the sexes. With this study we propose to analyse the spatial and temporal evolution of mortality in Cape Verde from 1995 to 2018, their temporal evolution by island, sex, age group, by group of causes of death. Additionally, to check for gender disparity in mortality rates and to project trends in overall mortality by sex and age group.

## Materials and methods

This is a quantitative, observational, ecological and time series study. All deaths that occurred in Cabo Verde from 1995 to 2018 were analysed. All stillbirths related to the analysed period were excluded from the study.

The database of the Integrated Disease Surveillance and Response Service of the National Health Directorate, Ministry of Health of Cabo Verde was used. The database is sourced from entries and death certificates issued by doctors and nurses in all Health Delegations at national level and sent to the Ministry of Health. It contains death records from 1995 to 2018 with information on causes and group of death according to ICD-10 (International Classification of Diseases - 10th revision), date of birth, date of death, sex, place of residence (zone, parish, county and island), place of death, information entered in the computer system and in Excel format.

### Data analysis

The analysis of time series to assess the spatiotemporal evolution of specific mortality rates [10] by island, sex, age group and cause of death, in the period from 1995 to 2018 were performed. Measures of association between group of causes of death and sex were done and a Regression analysis to verify the influence of the group of causes of death on the mortality rate [11].

Trends in mortality rates by categories were identified, being overall mortality by gender, mortality by gender and age group, mortality by gender and by island and by groups of causes of death with the greatest burden.

Based on the data obtained from the trends, a prediction of the trend in mortality by gender for the 2019–2025 horizon was made, using the Lee-Carter Mortality Projection Method [12]. It is a predictive model, which is based on historical information to predict the future, that is, it combines a demographic model for mortality with a time series model [ARIMA(p,d,q)], allowing extrapolation trends and age patterns of mortality [13].

Data processing and analysis are performed using the Statistical Package for Social Science (SPSS, version 26) and R (version 3.6.1) software, using the Demography package to implement the Lee-Carter method.

Statistical analysis is performed at a significance level of 0.05 and the data referring to Cabo Verde’s populations for each year are derived from Cape Verde’s demographic projections (2010–2030) [14].

### Ethics statement

The study does not involve risks of a physical, psychological, moral, intellectual, social or cultural nature. The project was approved by the National Ethics Committee for Health Research (CNEPS), Deliberation 74/2019 and by the National Data Protection Commission. Formal consent was not requested from family members, as they were anonymous data of death certificates available at the MS. The use of the anonymized database was authorized by the National Health Director, N/ref 275/INSP/2019. No form of consent was requested because the data provided by the ministry of health is anonymous.

Access to the anonymized database will be restricted to members of the research team. A code number was assigned to each element, thus ensuring data confidentiality.

## Results

### Distribution of deaths by island

From the analysis of mortality from 1995 to 2018, with N = 63,543 death records, it was found that Santiago islands presents the highest proportion of deaths 33,136 (52.1%), followed by São Vicente 10,941 (17.2%), Santo Antão 720 (11.3%), Fogo 4,903 (7.7%) and São Nicolau 2,591 (4. 1%). The first four islands are the most populous islands in Cabo Verde (2010 Census) and represent (58,772) 92.4% of mortality in the period 1995 to 2018.

Causes of death are grouped according to the International Classification of Diseases–ICD10 [15]. Diseases of the circulatory system (DAPC) occupy the first position with 15,741 (24.8%) deaths, followed by unclassified clinical signs and symptoms (SCNC), with 12,563 (19.8%), tumours with 6,558 (10.3%), infectious and parasitic diseases (IPAR) with 5,768 (9.1%), respiratory diseases (DARP), with 5662 (8.9%) and, lastly, trauma, poisoning and external causes (TECT), with 3,503 (5.5%) deaths.

The Tables 1 and 2 show the percentage distribution of deaths by year, island and cause of death. By analysing these data, it appears that diseases of the circulatory system show an increasing trend in all islands, except for the islands of Maio and Sal, which evolve in the opposite direction. It is the group of diseases that causes the most deaths on most islands (it reached values above 40% on the islands of São Nicolau and Brava). Regarding the group of unclassified clinical signs and symptoms, all islands show decreasing values. It represents the main group of causes of death on the islands of Santo Antão, São Nicolau, Maio and Fogo in the years 1995 to 2005, a situation that deserves further analysis.

**Table 1:** Distribution of mortality by group and cause of death (%) in the Barlavento Islands.

ISLAND	YEAR							
		DAPC	SCNC	TUMOURS	IPAR	TECT	DARP	OTHERS
Santo Antão	1995	13.2	44.88	3.63	4.62	8.91	7.26	17.49
	2000	24.51	28.46	11.86	5.14	3.95	3.56	22.53
	2005	16.52	27.09	12.35	4.78	3.98	8.37	23.9
	2010	25.16	19.68	13.87	5.48	6.77	7.1	21.94
	2015	23.91	26.4	11.49	3.42	4.04	11.18	19.57
	2018	25.43	8.67	14.74	6.65	7.23	18.79	18.5
São Vicente	1995	22.11	25.83	12.19	9.92	3.31	6.61	20.04
	2000	27.51	16.99	13.64	6.94	3.35	6.46	25.12
	2005	25.67	15.01	13.8	8.23	6.3	10.41	20.58
	2010	23.75	9.5	16.86	5.7	6.18	9.26	28.74
	2015	26.68	8.96	18.53	5.5	2.85	13.24	24.24
	2018	32.21	5.93	14.43	7.31	4.35	17.59	18.18
São Nicolau	1995	26.45	42.98	4.13	4.96	1.65	1.65	18.18
	2000	31.3	33.04	8.7	2.61	0	6.96	17.39
	2005	23.53	24.51	11.76	3.92	5.88	12.75	17.65
	2010	28.97	9.35	21.5	5.61	3.74	15.89	14.95
	2015	43.86	4.39	9.65	5.26	0.88	12.28	23.68
	2018	41.07	6.25	8.93	5.36	4.46	15.18	18.75
Sal	1995	27.59	17.24	5.17	5.17	10.34	6.9	27.59
	2000	16.95	11.86	10.17	15.25	13.56	3.39	28.81
	2005	20.29	11.59	8.7	7.25	20.29	7.25	24.64
	2010	19.59	10.31	17.53	9.28	9.28	10.31	23.71
	2015	18.87	10.38	13.21	5.66	12.26	10.38	29.25
	2018	22.22	5.13	10.26	11.97	3.42	17.09	29.91
Boavista	1995	20.83	20.83	20.83	12.5	4.17	4.17	16.67
	2000	31.58	10.53	10.53	15.79	0	21.05	10.53
	2005	30.77	23.08	3.85	11.54	15.38	3.85	11.54
	2010	27.91	6.98	9.3	6.98	16.28	9.3	23.26
	2015	32.65	6.12	8.16	8.16	6.12	22.45	16.33
	2018	37.5	14.58	8.33	6.25	4.17	8.33	20.83

Data source: Ministry of Health of Cabo Verde.

**Table 2:** Distribution of mortality by group of causes (%) in the Sotavento islands.

ISLANDS	YEAR	Causes of Mortality Group						
		DAPC	SCNC	TUMOURS	IPAR	TECT	DARP	OTHERS
Maio	1995	34.88	6.98	2.33	18.6	2.33	25.58	9.3
	2000	17.24	44.83	0.0	3.45	6.9	10.34	17.24
	2005	19.35	29.03	9.68	6.45	3.23	16.13	16.13
	2010	30.43	13.04	17.39	4.35	0.0	13.04	21.74
	2015	16.22	32.43	8.11	8.11	2.7	16.22	16.22
	2018	21.88	18.75	21.88	12.5	3.12	15.62	6.25
Santiago	1995	15.34	32.31	3.54	20.7	4.16	6.12	17.83
	2000	23.94	18.79	5.76	11.14	7.2	8.94	24.24
	2005	24.9	20.22	8.51	8.98	9.84	5.54	22.01
	2010	24.72	13.32	9.49	11.58	6.35	10.79	23.76
	2015	28.95	11.62	11.48	9.94	4.09	11.62	22.3
	2018	31.86	7.64	10.36	15.79	4.14	11.57	18.64
Fogo	1995	24.01	30.26	7.24	11.51	4.93	5.59	16.45
	2000	26.38	27.61	9.82	6.13	6.75	5.52	17.79
	2005	13.64	43.94	8.08	5.56	6.57	4.04	18.18
	2010	28.11	20	15.14	8.11	5.95	8.65	14.05
	2015	30.56	17.13	10.65	11.57	2.78	8.8	18.52
	2018	32.81	16.67	13.54	9.9	5.21	5.21	16.67
Brava	1995	19.7	30.3	1.52	27.27	1.52	0.0	19.7
	2000	22.45	18.37	10.2	10.2	8.16	12.24	18.37
	2005	40.0	20.0	10.0	8.0	4.0	2.0	16.0
	2010	41.03	7.69	7.69	12.82	2.56	10.26	17.95
	2015	26.32	18.42	21.05	13.16	0.0	2.63	18.42
	2018	16.36	27.27	10.91	12.73	1.82	12.73	18.18

Data source: Ministry of Health of Cabo Verde.

Deaths caused by tumours, with the exception of the island of Boavista, show a significant increase in all islands. In São Nicolau, Brava and Maio reached values above 21% in 2010, 2015 and 2018, respectively.

### Distribution of mortality by age group from 1995 to 2018

The age groups are grouped on a 5-year scale up to 95 years old and over, with the exception of those under one year and from 1 to 4 years old. The group younger than one year deserves to be highlighted with the highest number of deaths, 7,229 (11.4%).

The average age of death in 1995 was 50.65 years and the average was 63.00 years, in 2018 the average was 65.48 and the average was 73.00 years.

The number of male deaths is higher than that of females from zero years old to the age group of 70.00 to 74.00 years old. There is a higher female mortality from the age of 75.00 onwards (Table 3).

**Table 3:** Age distribution of deaths in the years 1995 to 2018.

	Year	Min.	1Qu.	Median	3 Qu.	Max.	Mean	SD
**Women**	1995	0.00	14.5	68.00	84.00	95.00	54.2	34.77
	2018	0.00	60.25	81.00	88.00	112.00	71.50	24.83
**Men**	1995	0.00	7.00	58.00	77.00	95.00	47.38	33.02
	2018	0.00	44.00	63.00	82.00	106.00	60.46	25.68
**Total**	1995	0.00	8.00	63.00	81.00	95.00	50.65	34.04
	2018	0.00	51.00	73.00	86.00	112.00	65.48	25.88

Data source: Ministry of Health of Cabo Verde.

### Distribution of mortality by sex and cause of death 1995 to 2018

**Fig 1:**
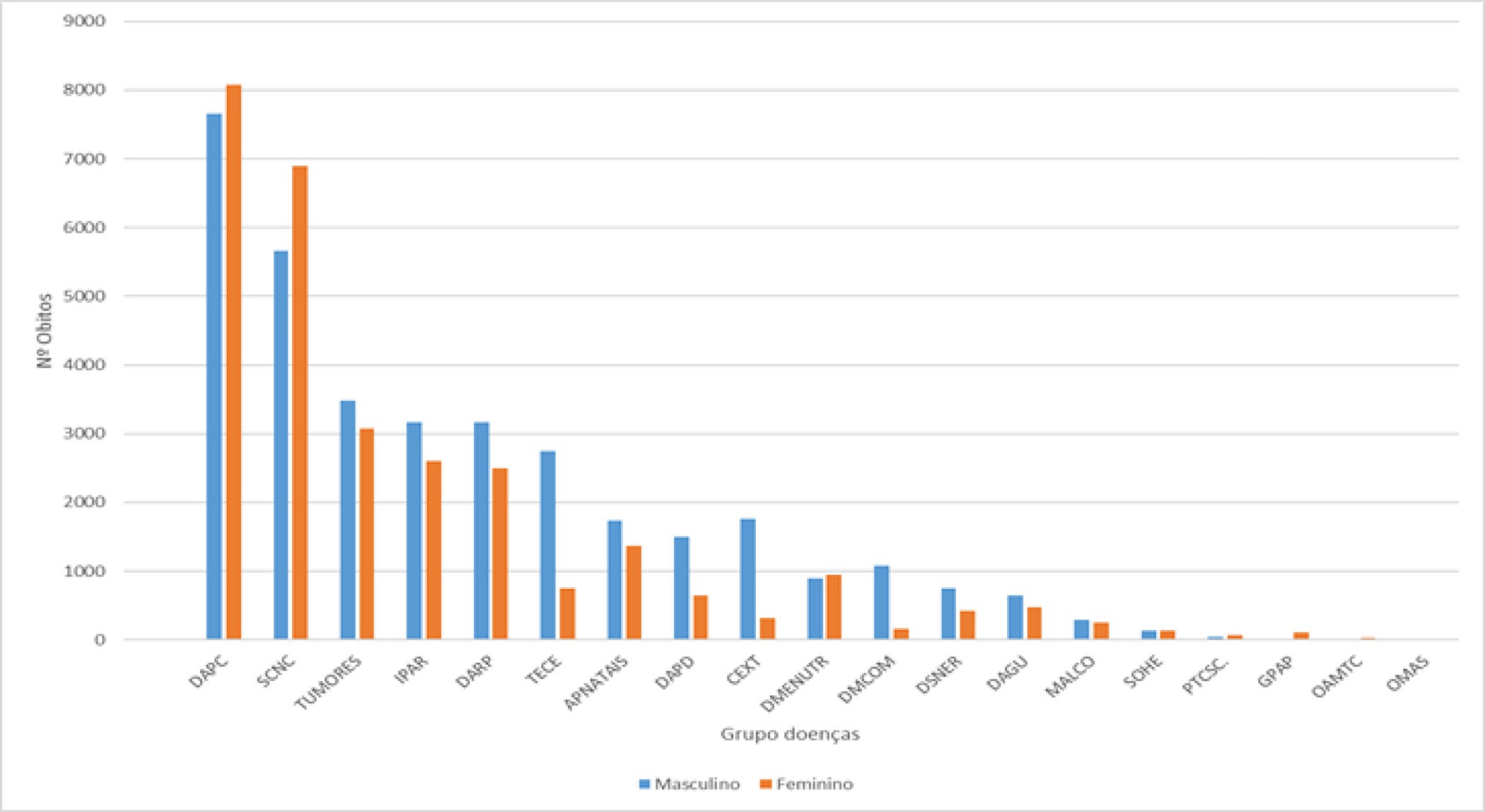
Distribution of deaths by sex and cause of death 1995 to 2018.

Males have a higher number of deaths in all death groups, with the exception of circulatory system diseases and unclassified clinical signs and symptoms, in which it is surpassed by females, as shown in Fig 1.

When considering deaths of both sexes, the causes shown in Table 4 were responsible for more than 75% of deaths each year.

**Table 4:** Distribution of mortality by year, sex and cause of death groups from 1995 to 2018.

YEAR	SEX	Causes of Death Group						
		DAPC	SCNC	TUMOURS	IPAR	TECT	DARP	OTHERS
1995	F	18.8	37.0	5.5	15.4	1.7	6.0	15.7
	M	16.9	27.4	4.9	17.1	7.0	6.4	20.3
	F+M	17.8	32.0	5.2	16.3	4.5	6.2	18.1
2000	F	27.1	25.2	9.6	9.5	3.6	7.9	17.3
	M	23.1	17.1	7.3	8.7	8.0	7.5	28.4
	F+M	24.9	20.8	8.3	9.1	5.9	7.7	23.3
2005	F	27.3	26.6	11.0	7.9	3.1	7.5	16.6
	M	20.6	18.2	9.0	7.8	12.8	6.4	21.2
	F+M	23.7	22.1	9.9	7.8	8.3	6.9	21.2
2010	F	31.0	16.1	13.8	9.3	2.7	10.0	17.0
	M	20.7	11.3	11.8	8.7	9.5	10.1	27.9
	F+M	25.2	13.4	12.7	8.9	6.5	10.1	23.1
2015	F	33.3	16.8	12.6	7.3	1.8	11.8	16.4
	M	24.3	10.2	12.7	8.7	5.7	11.6	26.7
	F+M	28.3	13.2	12.6	8.1	3.9	11.7	22.1
2018	F	37.1	10.0	11.6	11.2	1.7	13.4	15.0
	M	25.8	7.2	12.0	12.4	6.9	13.7	22.1
	F+M	30.9	8.5	11.8	11.8	4.5	13.6	18.9

Data source: Ministry of Health of Cabo Verde.

There is a greater weight in male mortality 34,733 (54.7%) throughout the study period, against 28,810 (45.3%) females.

The Table 4, show that DAPC increased throughout the study period and showed a higher percentage of occurrence in females. SCNCs have a higher percentage of females and show a decrease over the period under study. In relation to tumours, there is a higher percentage in women until the year 2010, becoming higher in men from that year onwards. The IPAR also occurred mostly in women until 2010, after becoming higher in men. The TECTs have a greater occurrence in men, had a growth from 1995 to 2005, to suffer a decrease until 2015. The DARPs, on the other hand, have evolved evenly in both sexes.

### Evolution of mortality from 1995 to 2018

The mortality trend from 1995 to 2018, with N = 63,543 death records, is decreasing from 1995 (4.3) to 2017 (3.9) and increases in the last year of the 2018 study (4.5).

### Evolution of mortality per island from 2010 to 2018

In order to compare the level of mortality between the islands, the standardized mortality rate due to all causes, by age in each of the islands, was calculated for the period 2010 to 2018. It was chosen to include from 2010 onwards by lack of Census data from previous years.

The Table 5 shows the mortality rates in Cabo Verde from 2010 to 2018, the period for comparing mortality between islands. It is noteworthy that there is a positive evolution of mortality on the island of Sal (46.4), which had a rate above the national average level (47.33), but it has been decreasing until, as of 2014, it has been below that level. The islands of Boa Vista (37.37), Fogo (43.69), Maio (39.14), Santiago (45.81) and Sal (46.4) have an average rate lower than the national level (47.33), while for the islands of São Nicolau (70.76), Brava (57.81), Santo Antão (52.65) and São Vicente (49.11) the opposite happens, the average is higher than the national one (47.33). However, from 2015 onwards, the mortality rate on the island of São Vicente shows a converging trend towards the national level.

**Table 5:** Total mortality rate (per 10 thousand) per island (2010–2018).

Islands	2010	2011	2012	2013	2014	2015	2016	2017	2018	Média
Nacional / Cabo Verde	**48.19**	**49.95**	**50.8**	**47.65**	**47.09**	**48.49**	**44.72**	**42.21**	**46.88**	**47.33**
Santo Antão	54.54	58.03	55.34	57.75	51.1	51.27	48.34	43.31	54.13	52.65
São Vicente	50.59	51.24	56.74	50.09	50.09	48.91	45.17	41.78	47.42	49.11
São Nicolau	80.77	69.41	74.7	78.37	67.28	79.37	62.51	59.22	65.23	70.76
Sal	60.34	49.75	50.57	50.61	43.04	41.16	34.63	42.22	45.26	46.40
Boa Vista	45.51	37.4	33.41	37.81	28.69	44.74	32.92	45.12	30.72	37.37
Maio	28.67	46.99	38.31	43.7	49.02	41.93	40.39	30.65	32.56	39.14
Santiago	46.57	48.73	48.21	44.84	45.94	47.89	43.98	40.31	45.82	45.81
Fogo	42.41	40.54	48.43	39.57	41.18	48.71	43.71	46.12	42.5	43.69
Brava	53.66	60.46	57.93	53.53	66.23	53.08	48.02	54.57	72.8	57.81

Data source: Ministry of Health of Cabo Verde.

**Fig 2:**
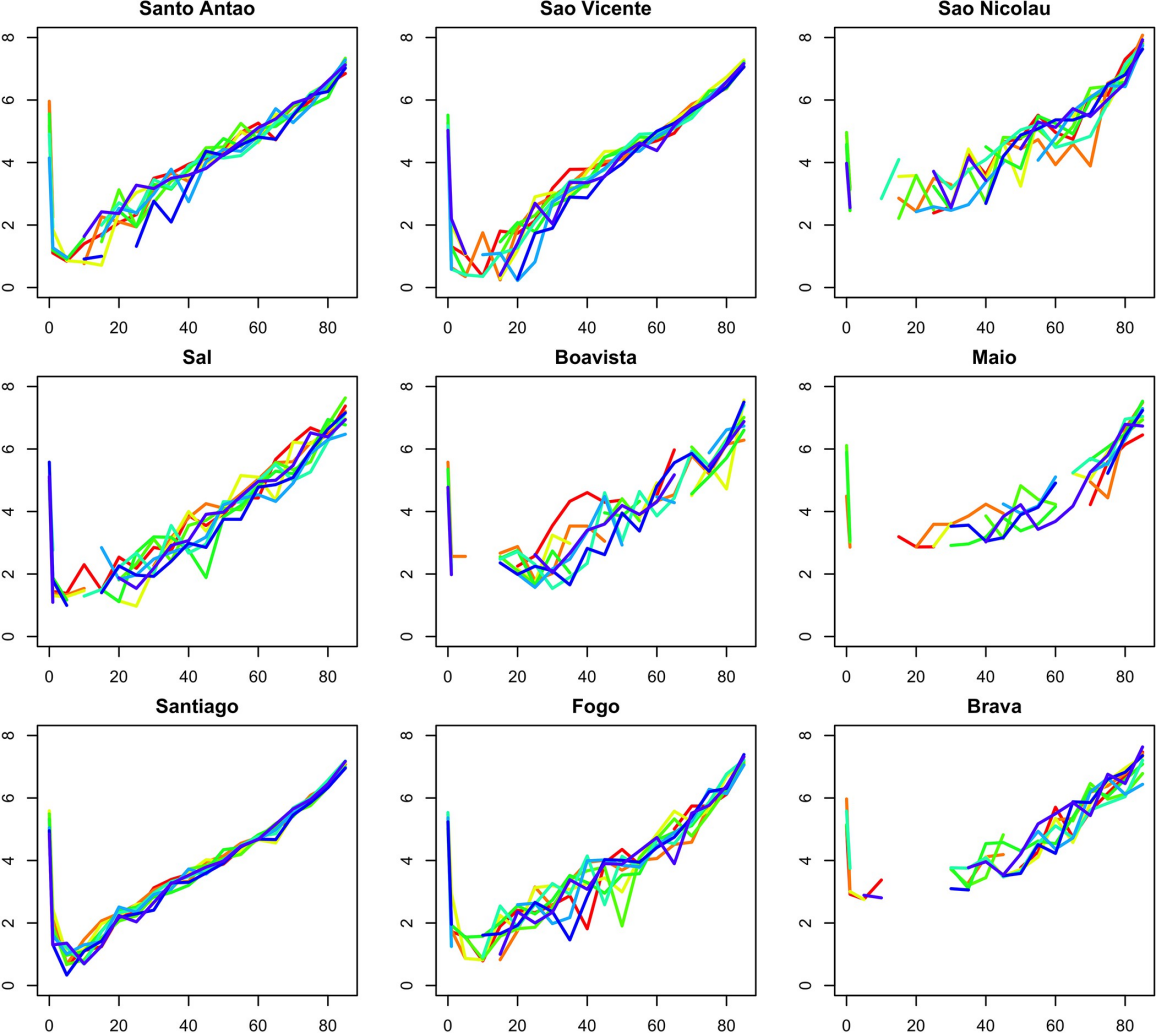
Graphic evolution of specific annual mortality (2010 to 2018) by island and age.

The graph in Fig 2 shows the mortality profile on each island, through the specific mortality rate by age group. There is an increase in the mortality rate on all islands, which increases with age. However, there are fluctuations across the age groups, especially on the islands of São Nicolau, Boa Vista and Fogo.

### Evolution of mortality by age group 1995 to 2018

**Fig 3:**
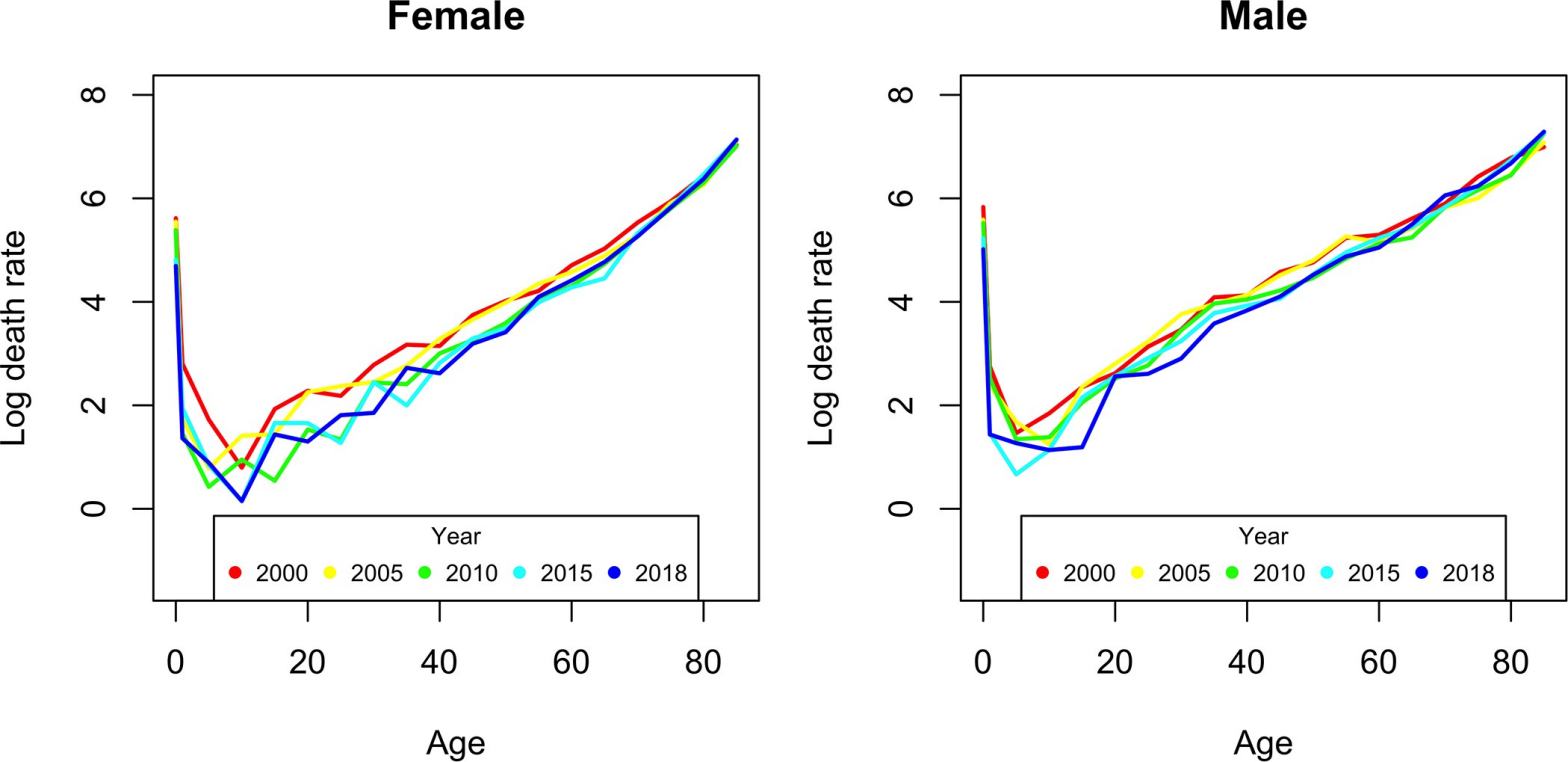
Specific mortality rate (per 10 thousand) by age and sex in 2000, 2005, 2010, 2015 and 2018.

The age and sex-specific mortality rate highlights a high infant mortality rate (under one year of age) and a decrease in this rate for children under ten over the study period and for both sexes. There is a decrease in the mortality rate from 2000 to 2018, in men aged less than or equal to 60 years. In women, despite a downward trend, there seems to be an oscillation in the years 2010 to 2018 (Fig 3).

The specific mortality rate for women aged 50–54 years is similar to that for men aged 35–39 years, suggesting an early mortality rate for men compared to women (15 years differential), which corroborates the results from Lenart, et. al. (2019) [16].

**Fig 4:**
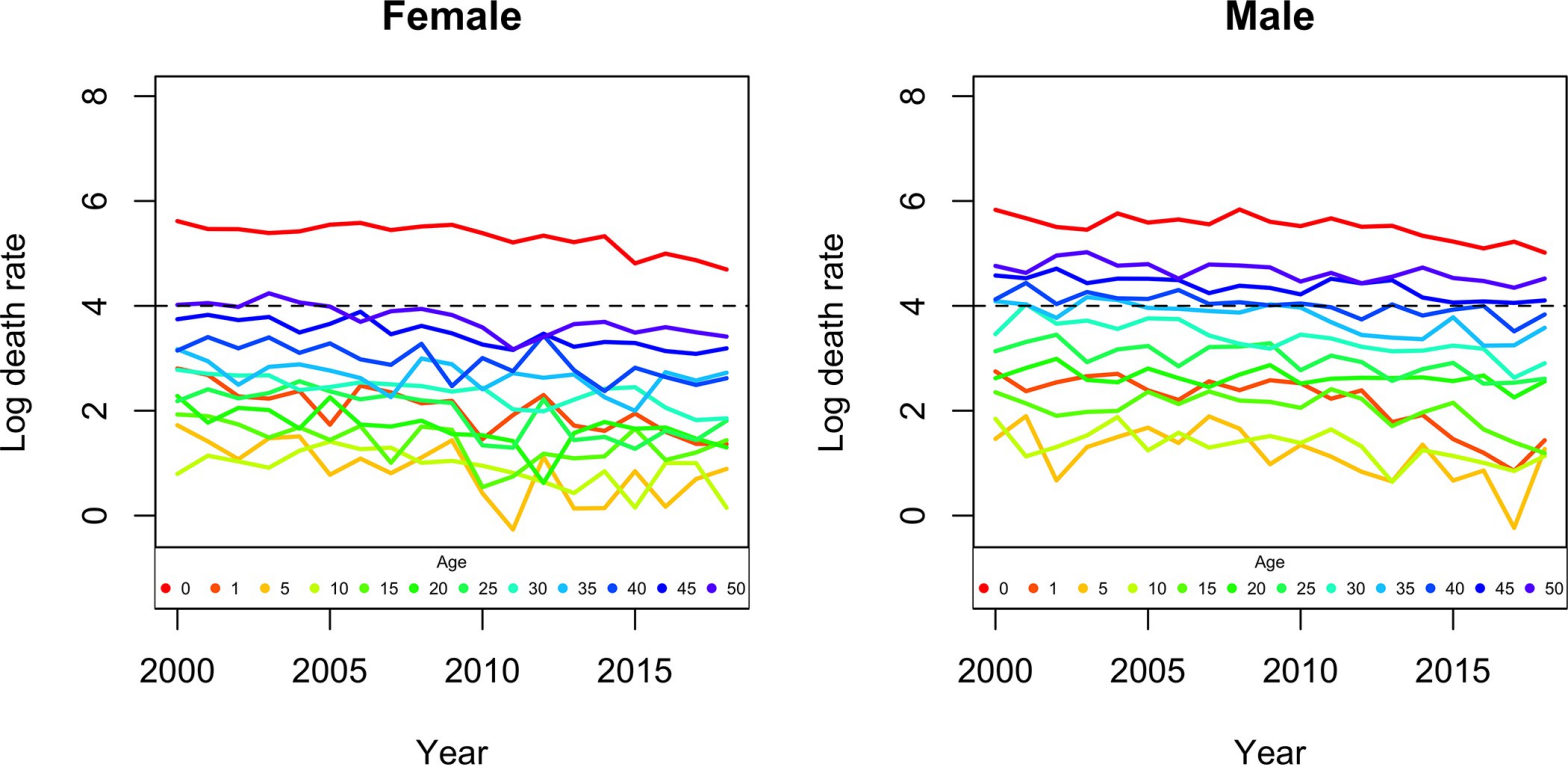
Age-specific mortality rate (0 to 50 years) and sex.

Mortality rates in children under the age of five show a downward trend over the years in both sexes. For female children under the age of one year, the rate decreased from 231.5 to 97.5 (per 10 thousand) representing a decrease of 58.8% from the year 2000 to 2018. In the age group 1 to 4 years it decreased from 16.5 to 3.9, a reduction of 76.3%. For male children under one year and in the same period, the rate decreased from 280.32 to 132.16, translating into a decrease of 52.8% and for the age group from 1 to 4 years, there was a decrease from 15.6 to 4.2, or be a decrease of 73.0% (Fig 4).

### Evolution of mortality due to death from 1995 to 2018

The trend of standardized mortality due to diseases of the circulatory system is decreasing from 2000 to 2010 and increasing from 2010 to 2018. The mortality rate in men is higher than in women, and the difference in mortality between the sexes shows an increasing trend during the period under study. In 2000 the difference was 5.2 (18.4–13.2) and in 2018 it was 4.1 (17.1–13).

**Fig 5:**
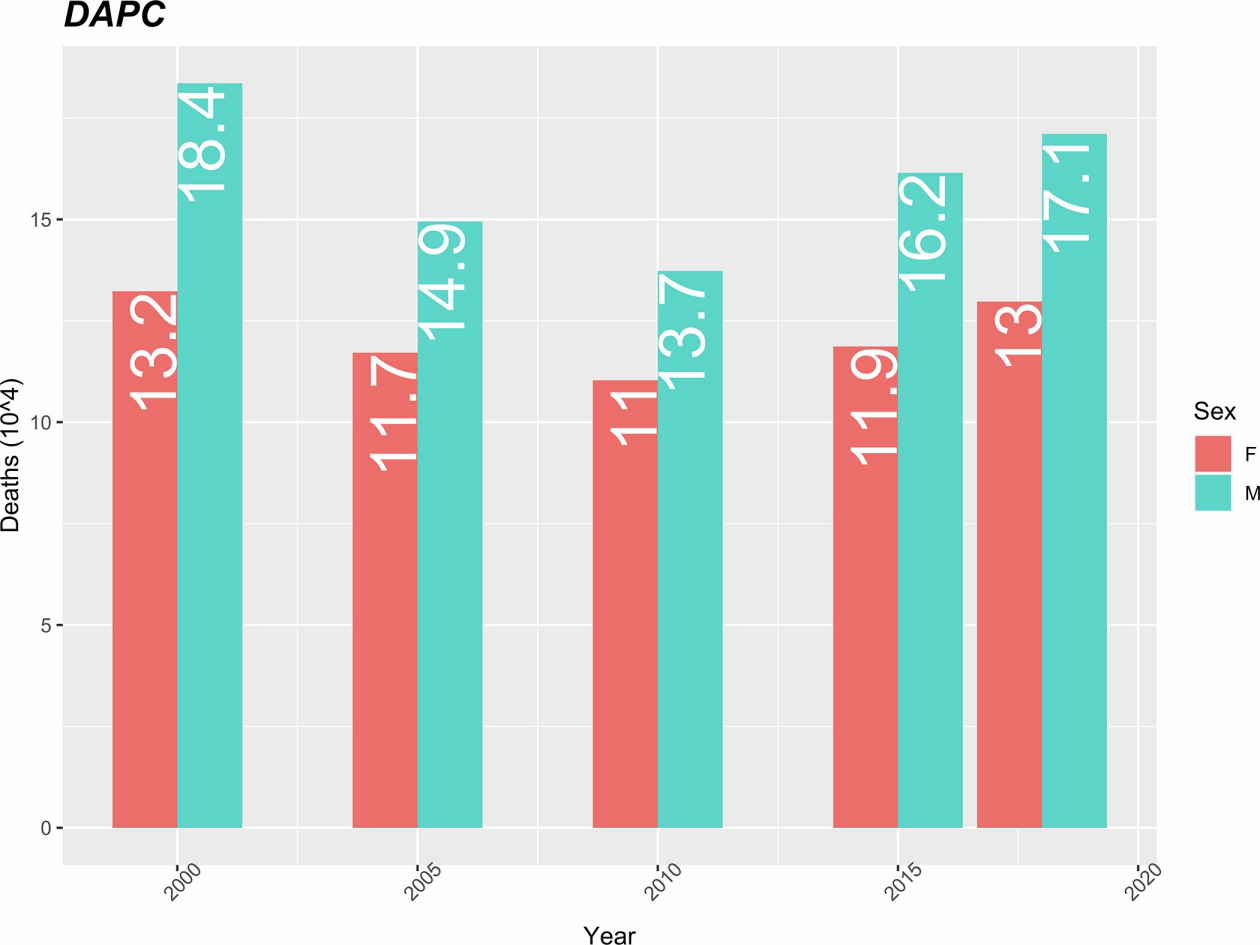
Mortality rate due to DAPC (10 thousand) standardized by age.

In percentage terms, diseases of the circulatory system represent the second leading cause of death in women and the third in men, 18.8% and 16.9% respectively, in 1995. They constitute the leading cause of death in 2000 for both women and men, causing respectively 27.1% and 23.1% of total deaths and continue in that position until 2018, when they are responsible for 37.1% of total deaths in women and 25.8% in men, it has the highest mortality rate among the groups of causes considered throughout the period analysed (Fig 5).

**Fig 6:**
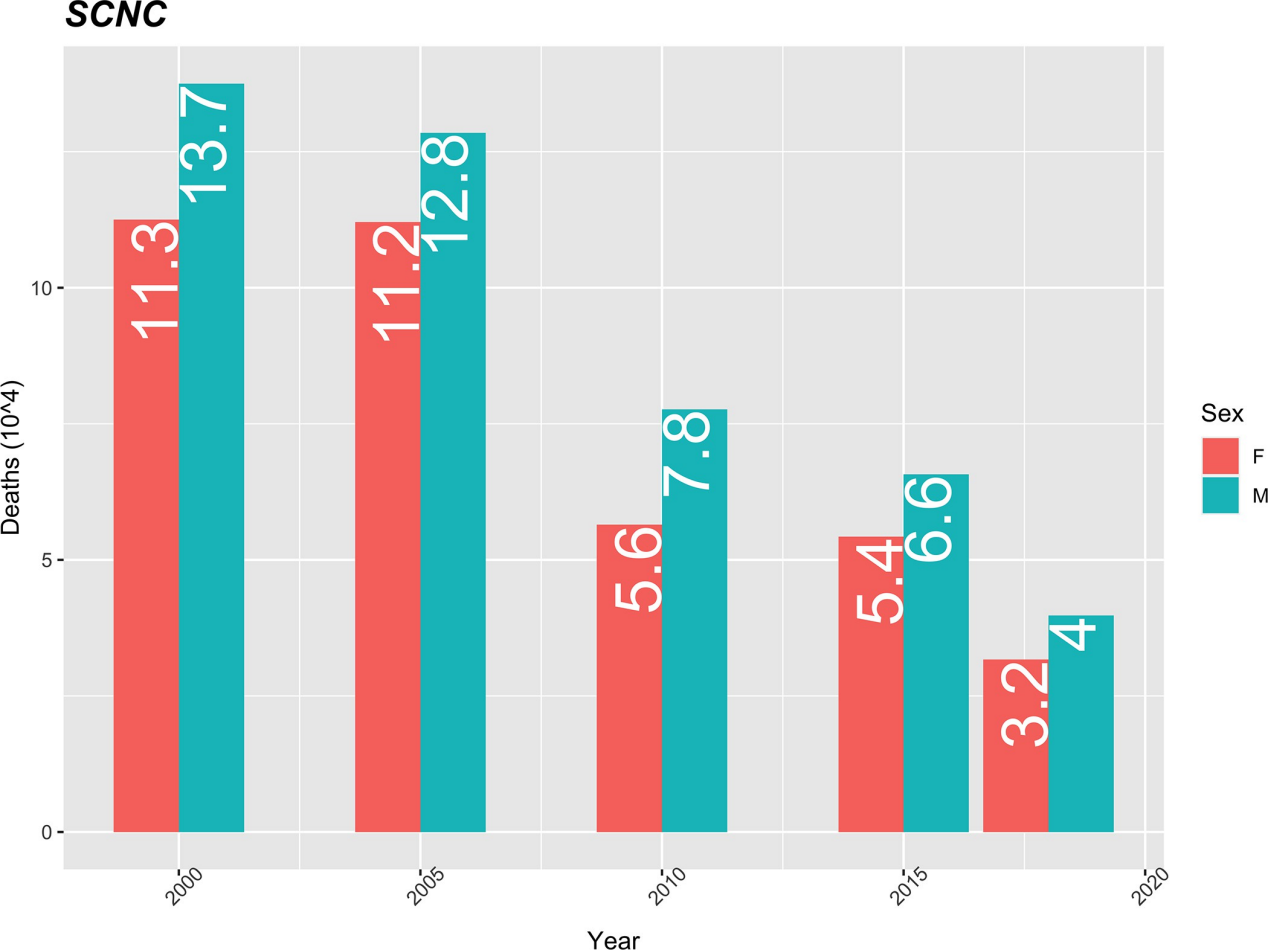
SCNC mortality rate (10 thousand) standardized by age, using the population of Cape Verde in 2010 as the standard.

The mortality rate from unclassified clinical symptoms is higher in men. These rates show a downward trend over time in both sexes. In 2000, the difference in rates between the sexes was 2.4 (13.7–11.3) and in 2018 it was 0.8 (4–3.2). This same trend is verified when analysed in percentage terms (Fig 6).

In the mortality rate from tumours in men, there is an increasing trend from 2000 to 2015, having decreased slightly in 2018. However, the mortality rate due to this cause in men was always higher than in women over the years. This rate remained around 5 (10 thousand) with slight fluctuations.

**Fig 7:**
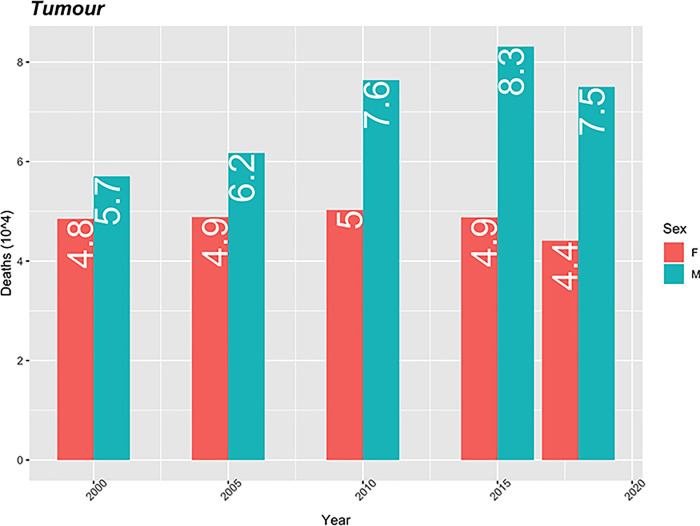
Tumour mortality rate (10 thousand) standardized by age, using the population of Cape Verde in 2010 as the standard.

In men, the rate increased from 5.7 in 2000 to a maximum of 8.3 in 2015. It should be noted that the difference in this rate between the sexes increased over time, with values of 0.9 in 2000 and 3.1 in 2018 (Fig 7).

**Fig 8:**
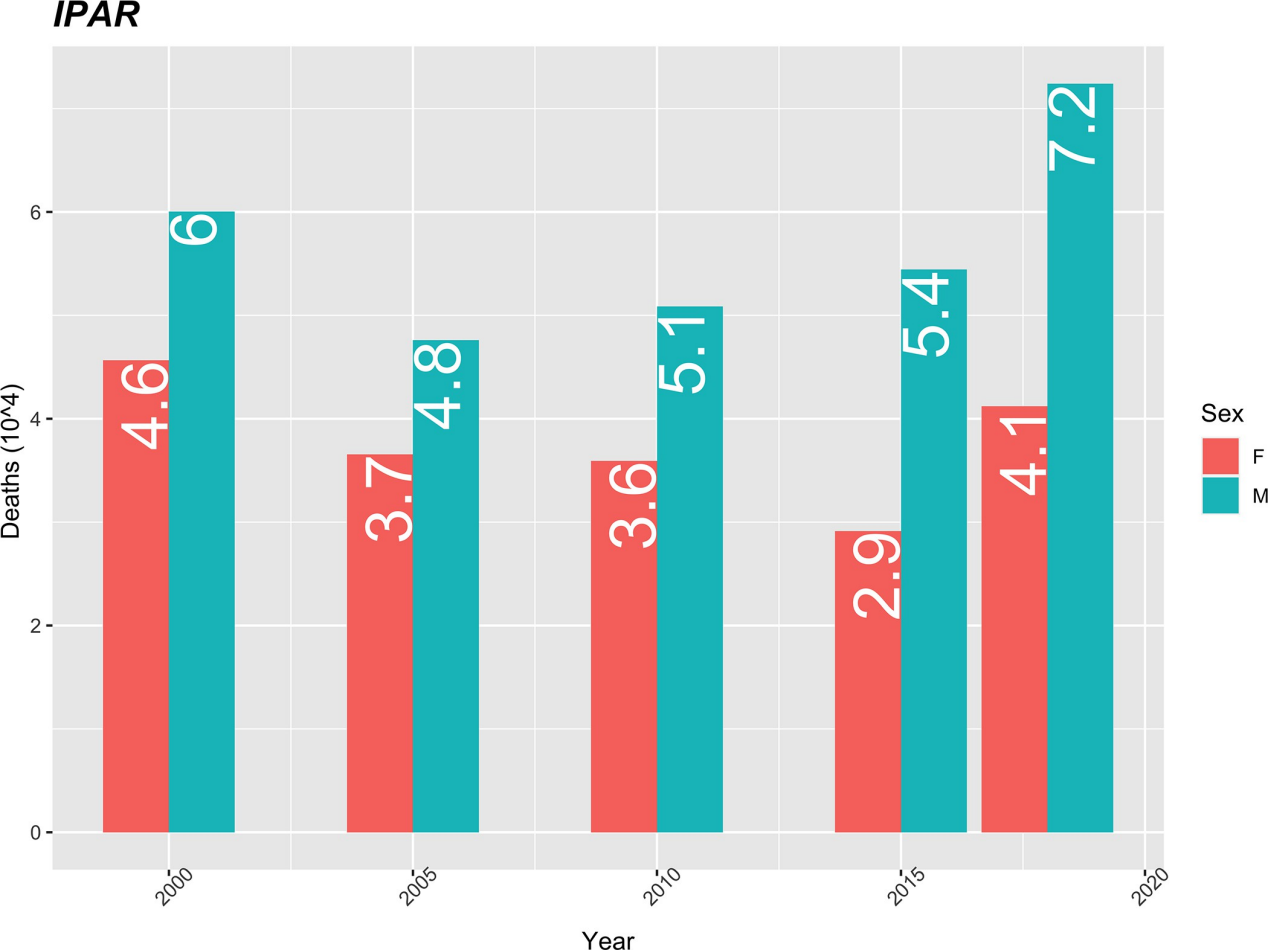
Mortality rate per IPAR (10 thousand) standardized by age, using the population of Cape Verde in 2010 as the standard.

The number of deaths caused by infectious and parasitic diseases is higher in men throughout the study period and shows an increasing trend from 2005 to 2018 (increases from 4.8 in 2005 to 7.2 in 2018). In women, the mortality rate shows a downward trajectory until 2015, it decreases from 4.6 in 2000 to 2.9 in 2015, having increased to 4.1 in 2018 (Fig 8).

**Fig 9:**
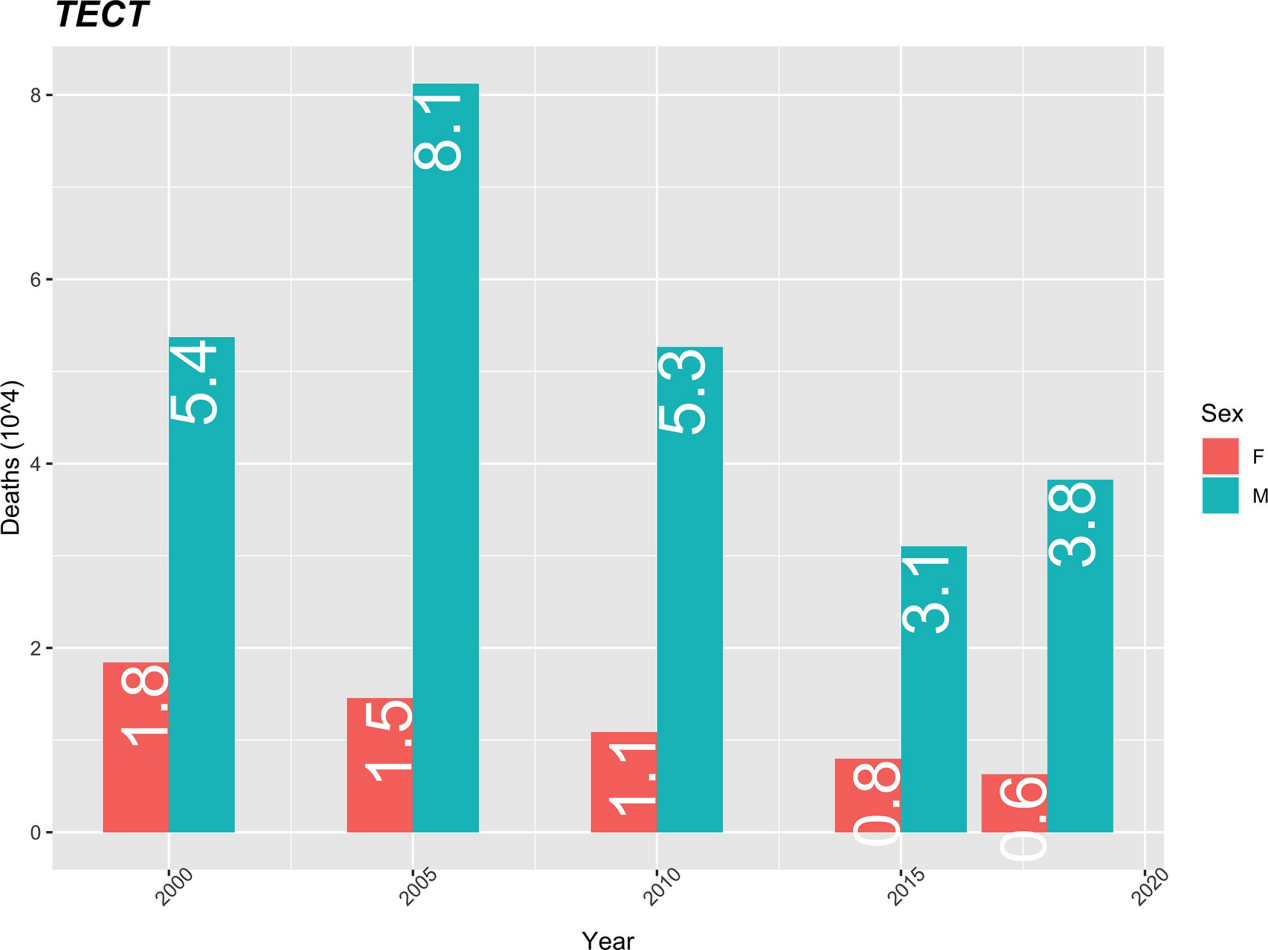
Mortality rate per TECT (10 thousand) standardized by age, using the population of Cape Verde in 2010 as the standard.

The mortality rate from injuries, trauma, poisoning and external causes ranks sixth among the causes of death. This rate is higher among males when compared to females, but shows a decreasing trend. Male mortality peaks in 2005 at 8.1 and declines again to 3.8 in 2018, representing a 53% reduction. From 2005 to 2018 the mortality rate for men is on average four (4) times the mortality rate for women. The female mortality rate from these causes, in addition to being low, shows a decreasing evolution over the entire period (Fig 9).

**Fig 10:**
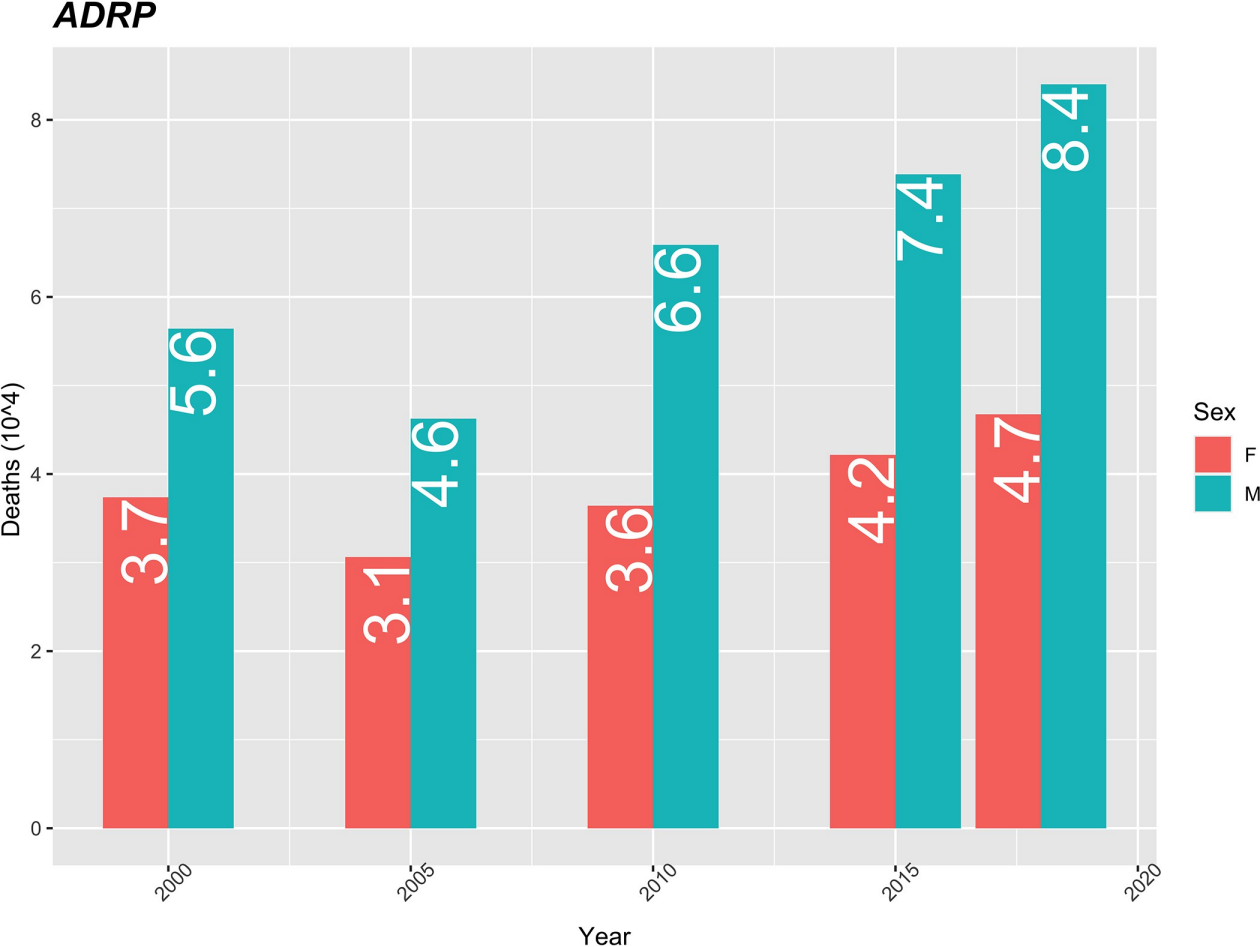
Age-standardized mortality rate due to DARP (10 thousand), using the population of Cape Verde in 2010 as the standard.

Respiratory system diseases represent 8.9% of the cases and occupy the 5th position in the group of causes of death during the analysed period. They show a growing trend for both sexes, as of 2005, and the male mortality rate is always higher than that of the female. The male mortality rate increased from 4.6 in 2005 to 8.4 in 2018, which represents an increase of 82%. The rate increase in women was 51% over the same period (Fig 10).

### Projection of Mortality for the Period 2019 to 2025

The Lee-Carter method is applied to global specific mortality rates (both sexes) and also distributed by sex, as mortality is different between men and women. The explained variation was 58.4% for females, 64.4% for males and 72.2% for the total (both genders).

**Fig 11:**
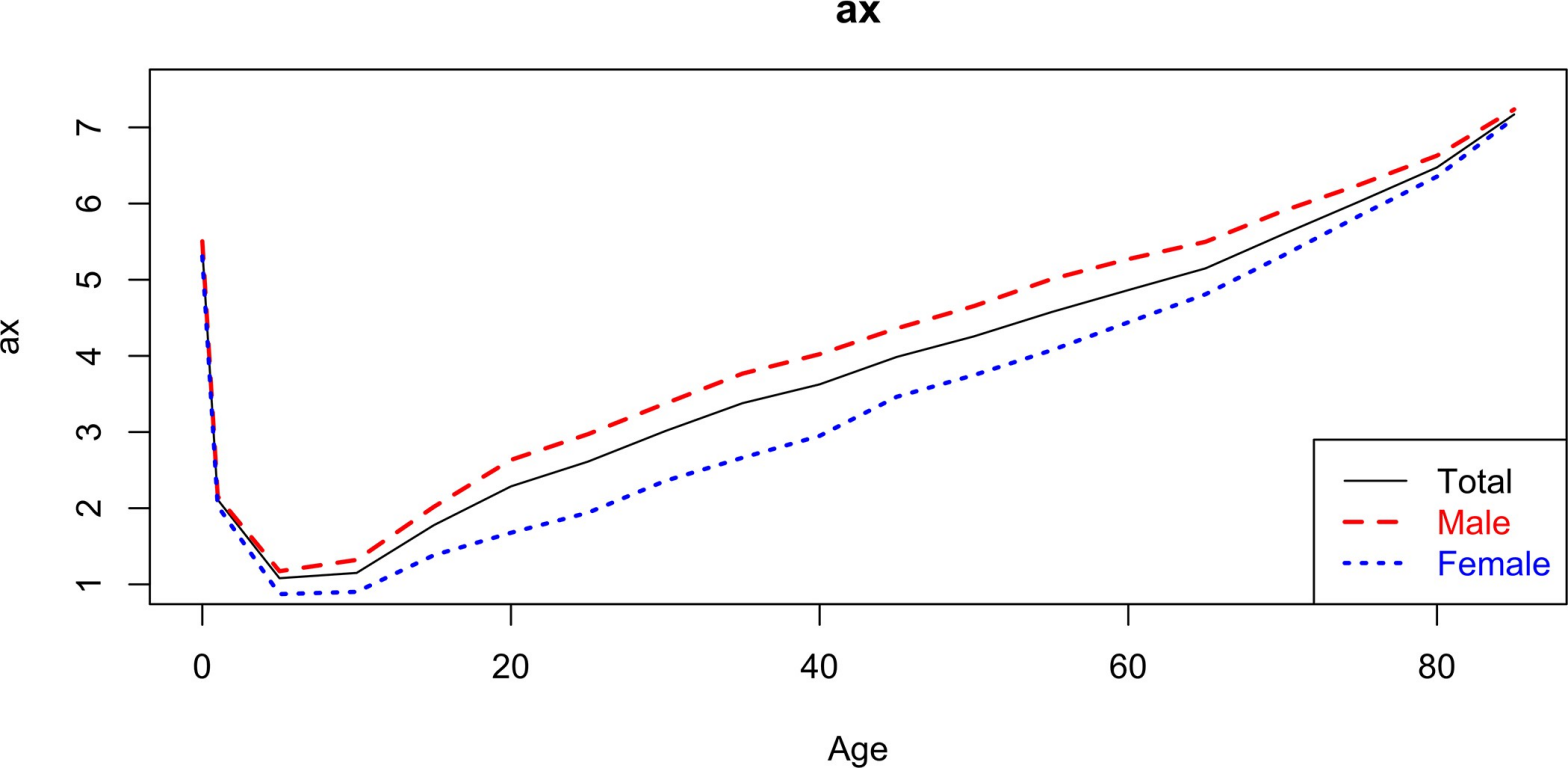
Estimate of ax, from the Lee-Carter model for total and by sex—(2000–2018).

The Lee-Carter ax method (Fig 11) expresses the average of mortality rates for each age group x, over time (t). It appears that the male mortality rate is clearly higher than the female one. Furthermore, it is visible that the male mortality rate curve has a greater slope between the range of 10 to 20 years, which can be confirmed through the values of ax in Table 6.

**Table 6:** ax and bx of the Lee-Carter model, by age group and by sex.

AGE	FEMALE		MALE		TOTAL	
	ax	bx	ax	bx	ax	bx
0	5.307	0.051	5.504	0.061	5.414	0.066
1–4	2.028	0.097	2.155	0.170	2.112	0.140
5–9	0.872	0.126	1.173	0.135	1.081	0.114
10–14	0.902	0.044	1.321	0.073	1.150	0.066
15–19	1.383	0.079	2.018	0.083	1.777	0.070
20–24	1.677	0.048	2.634	0.024	2.287	0.034
25–29	1.942	0.105	2.973	0.075	2.613	0.085
30–34	2.359	0.057	3.375	0.088	3.013	0.080
35–39	2.663	0.040	3.767	0.085	3.379	0.066
40–44	2.950	0.061	4.022	0.051	3.626	0.050
45–49	3.463	0.063	4.361	0.045	3.986	0.046
50–54	3.748	0.063	4.654	0.040	4.256	0.042
55–59	4.072	0.029	5.006	0.032	4.574	0.029
60–64	4.441	0.043	5.271	0.019	4.864	0.033
65–69	4.809	0.41	5.498	0.008	5.149	0.027
70–74	5.314	0.015	5.901	-0.0002	5.594	0.013
75–79	5.843	0.0001	6.249	0.012	6.029	0.011
80–84	6.354	0.016	6.629	0.0002	6.475	0.014
85+	7.119	0.021	7.236	0.0002	7.170	0.013

Data source: Ministry of Health of Cabo Verde.

**Fig 12:**
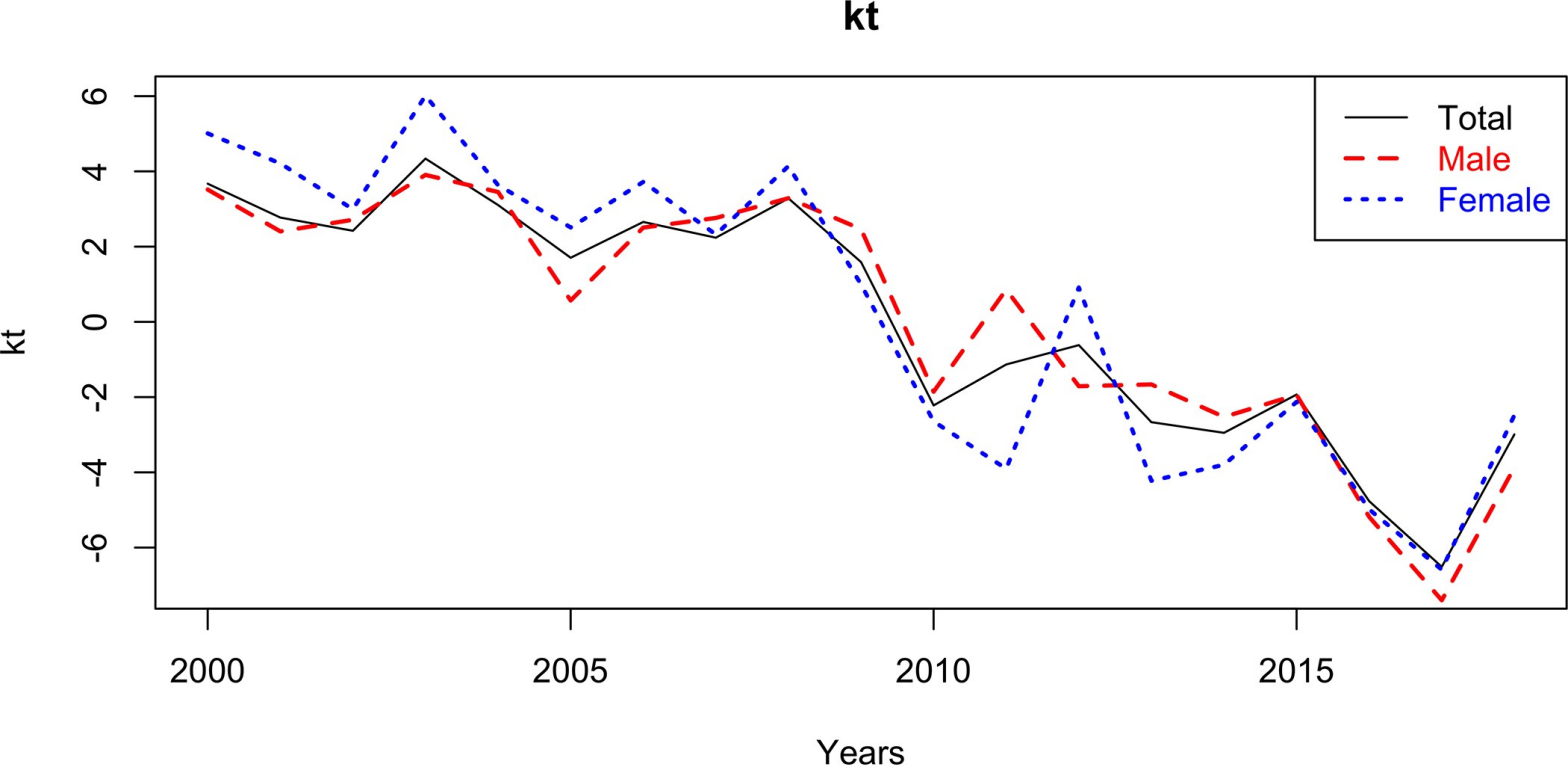
Adjusted kt estimate, from the Lee-Carter model for males, females and both genders (2000–2018).

The *k_t_* estimates (Fig 12) allow us to identify the trend in the level of mortality over time. Although with many fluctuations, overall mortality tends to decrease over time. It is noteworthy, however, an increase in mortality in 2018.

**Fig 13:**
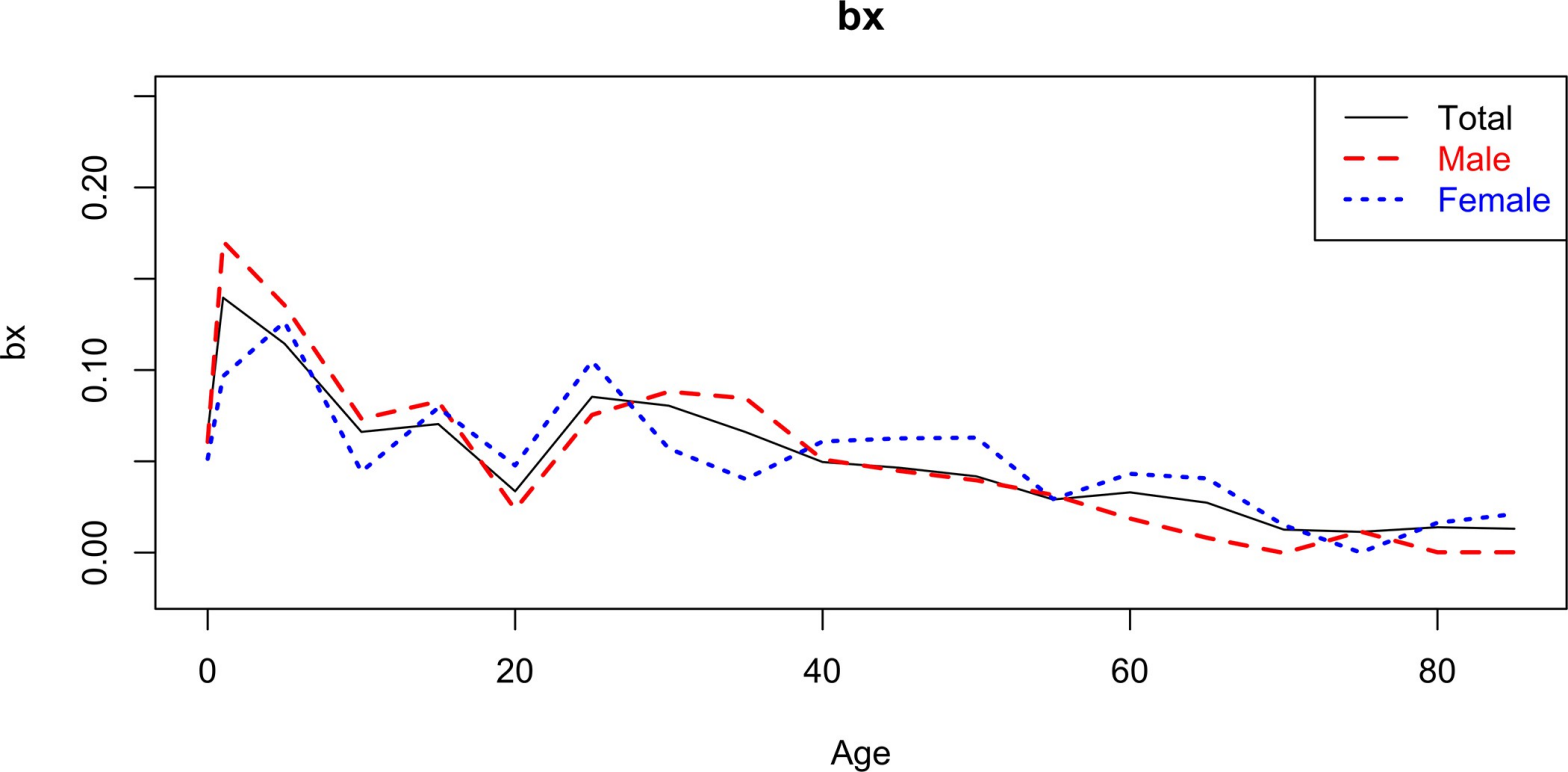
Estimate of bx, from the Lee-Carter model by sex for the total (2000–2018).

The coefficient bx (Fig 13) measures the sensitivity of change in mortality at age x as a function of kt, the higher the value of bx, the more sensitive is the mortality in this age group in response to the general variation in the level of mortality.

In age groups under 35 years, there is greater sensitivity to the variation in the kt coefficient, and in males there is greater sensitivity to the variation in kt for the age group below 20 years (Table 6). (In other words, there is a greater variation in the average level of mortality in this age group in face of fluctuations in the global mortality trend).

The average level of specific mortality for men aged 50–54 years is similar to that for women aged 35–39 years.

**Fig 14:**
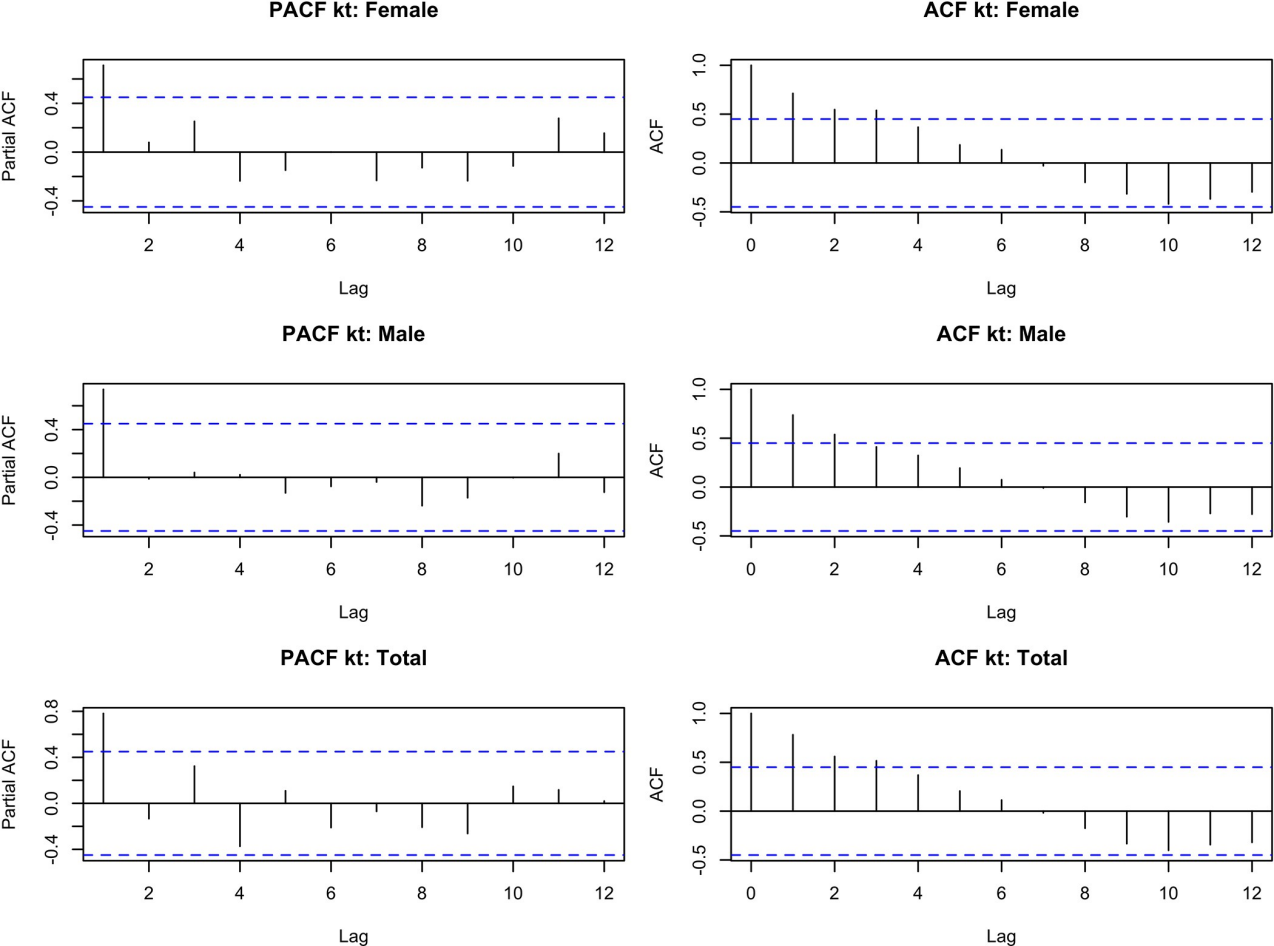
Partial autocorrelation function (PACF) and autocorrelation ACF) of kt estimated in the Lee-Carter model by sex.

In order to identify the appropriate ARIMA model to predict future values for k_t, graphical inspections and normality tests of the residuals and the Ljung Box test are used to assess the self-correlation in the residuals. We can see from the autocorrelation and partial autocorrelation functions (Fig 14) that this is a non-stationary process.

The model that best fits the given series was automatically selected, using auto.arima, based on one of the AIC or BIC criteria. For females and for the total, ARIMA (0.1.0) was chosen, while for males the ARIMA (0.1.1) with drift. However, from the analysis of the ACF and PACF graphs, some models were tested, making it possible to identify (based on the AIC criterion) models different from those selected by auto.arima.

**Fig 15:**
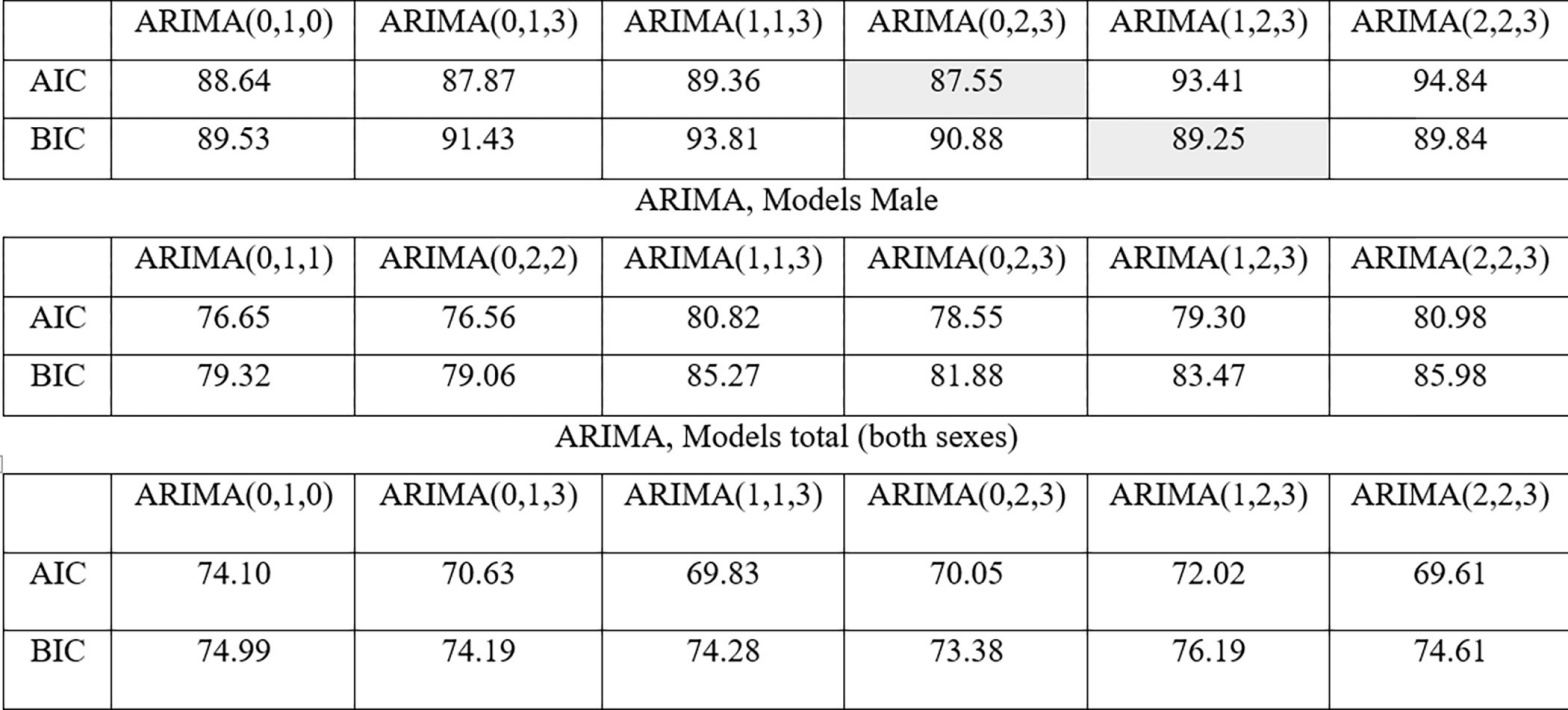
ARIMA models, male, female and both genders.

For the prediction of widehatkt values, the models that presented the lowest value of the Akaide criterion were selected. Thus, for females, ARIMA (0.2.3) was chosen with AIC = 87.55 and ARIMA (1.2.3) with BIC (89.25), and for males, the ARIMA model (0.2,2), by the criteria AIC = 76.56 and BIC = 79.06 (Fig 15). For the total (both sexes) the selected model was ARIMA (2,2,3), whose AIC value = 69.61, while for BIC = 73.38 the selected model would be ARIMA (0.2,3). The adequacy of the models was confirmed by the normality test of the residuals, and by the Ljung Box test, which confirmed the absence of self-correlation in the residuals.

**Fig 16:**
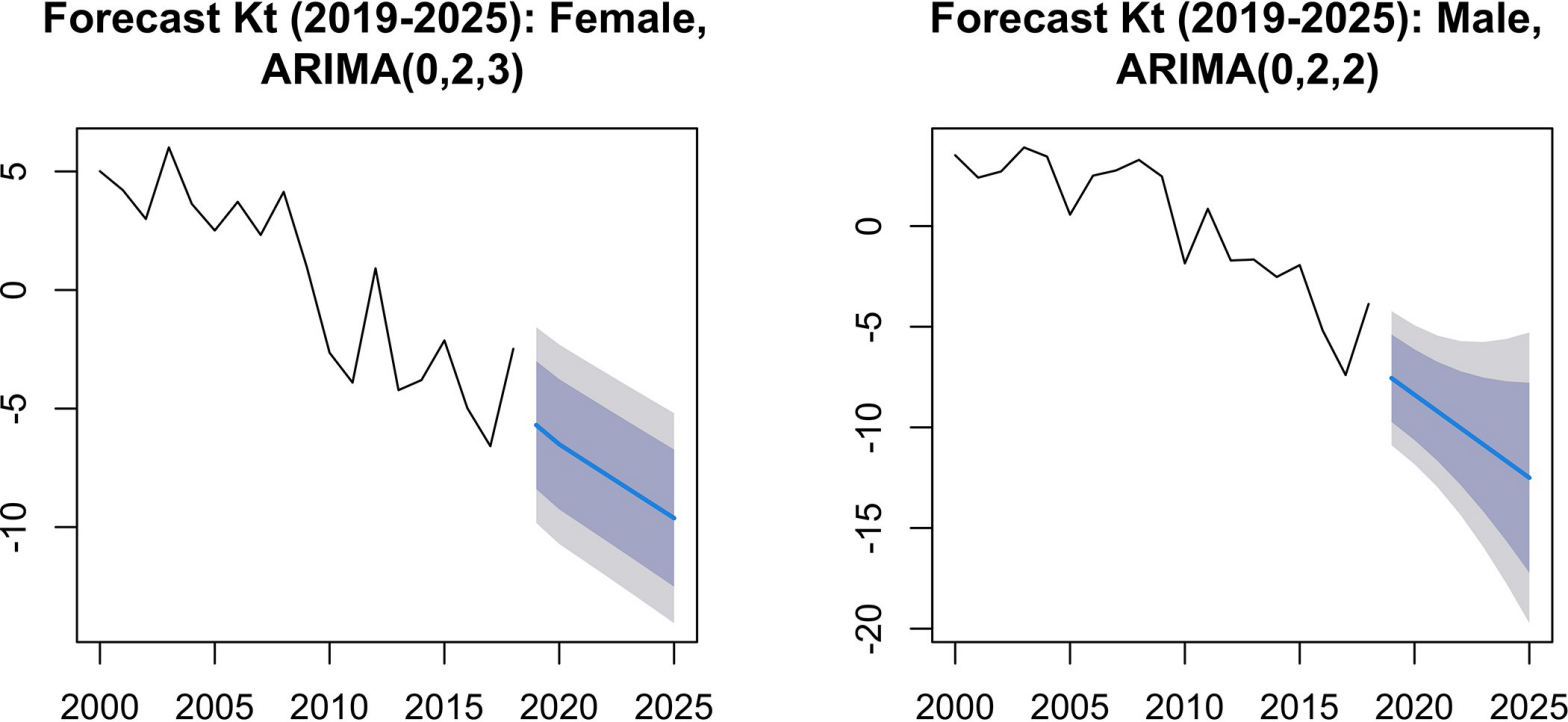
Forecast of kt, (2019–2025) by the ARIMA model (0,2,3) for females and ARIMA (0,2,2) for males.

**Fig 17:**
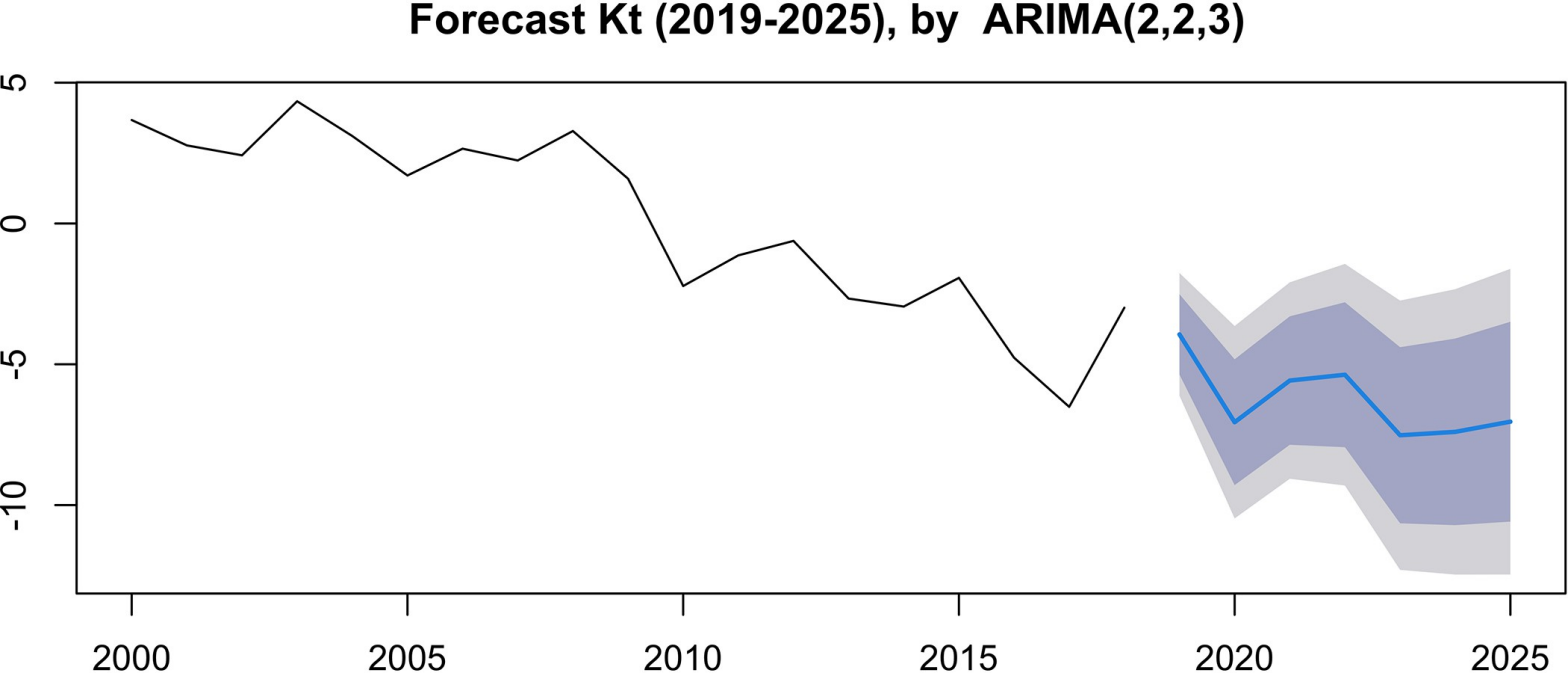
***k_t_***, estimated in both sexes (2019–2025), by the ARIMA model (2, 2, 3).

**Fig 18:**
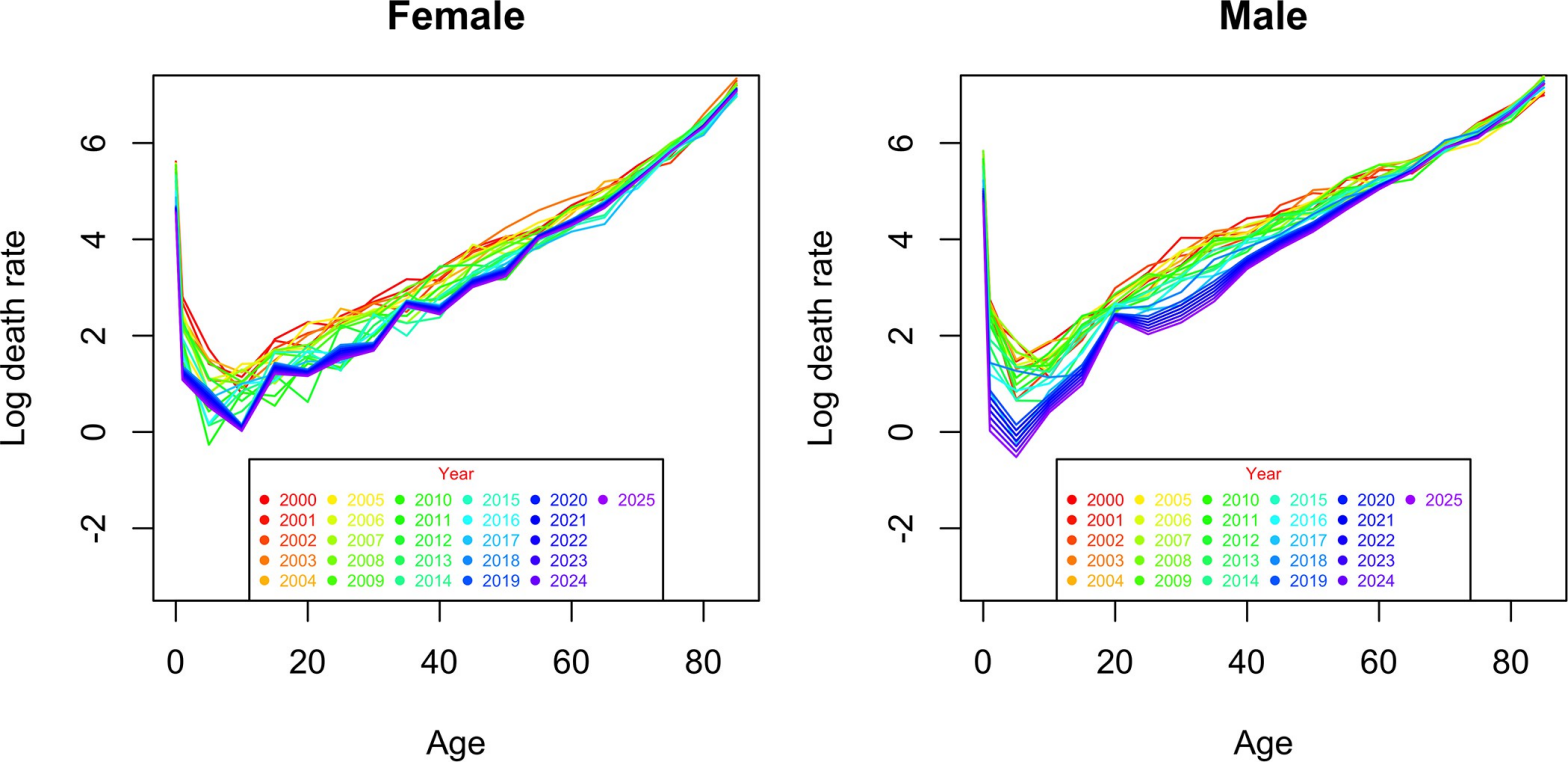
Observed (2000–2018) and projected (2019–2025) logarithmic mortality by the male and female Lee-Carter method.

**Fig 19:**
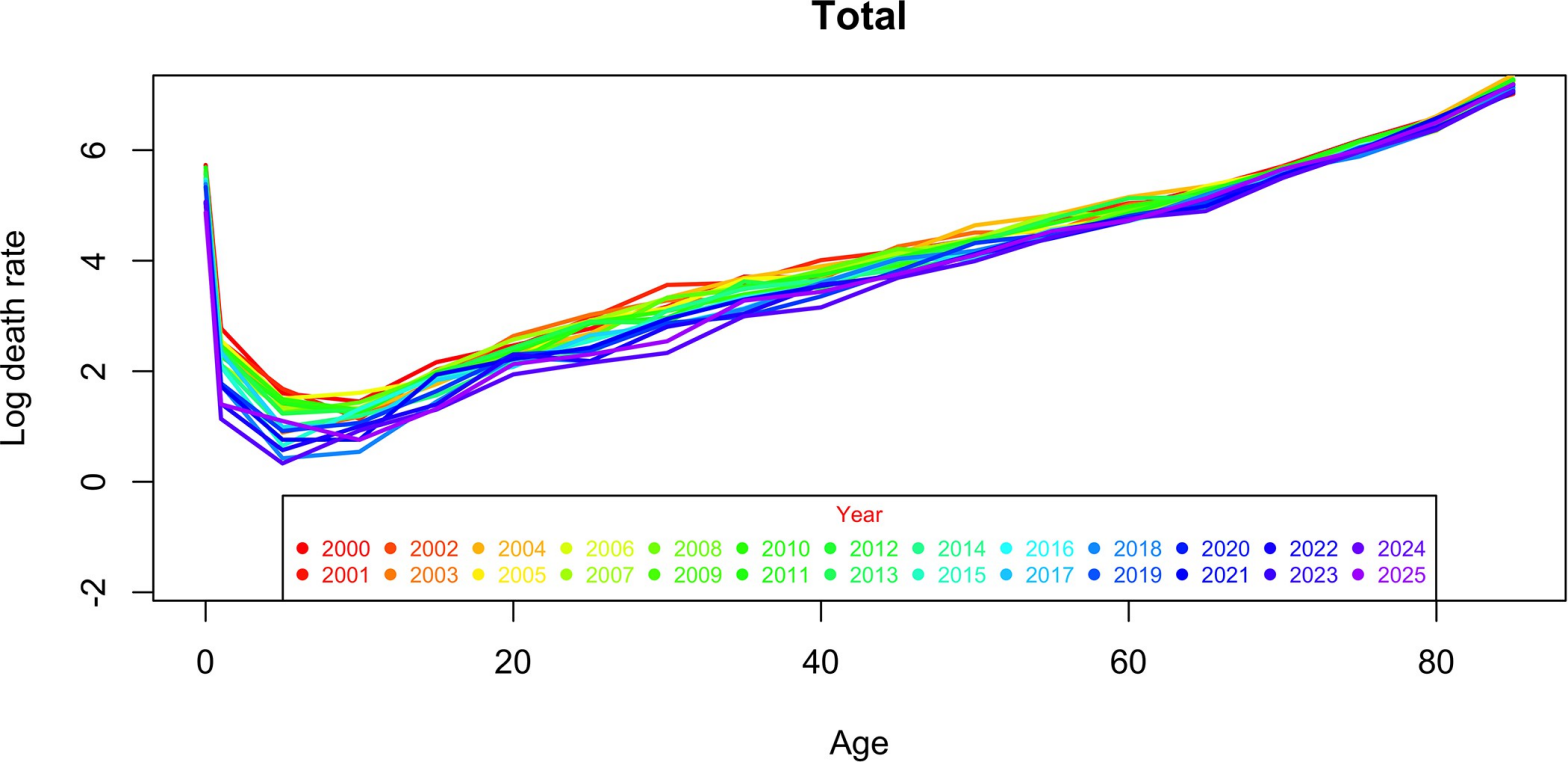
Total logarithmized mortality observed (2000–2018), and projected (2019–2025) by the male and female Lee-Carter method.

Figs 16 and 17 present the predictions for the mortality trend index using the selected ARIMA models, at 80% and 95% confidence intervals (Table 7, 8 and 9). The range widens as the forecast horizon widens (estimates become more uncertain). Using the estimated values of a_x, b_x and projected values of k_t, mortality is predicted for the period 2019 to 2025. Analyzing the graphs in Figs 18 and 19, in general terms, a downward trend is predicted of the logarithmic mortality rates in Cape Verde, with the exception of the age group above 65 years, which is in accordance with the pattern observed during the study period. A greater gain is expected in the under-20 age groups, with males appearing to be the ones who will benefit most from the reduction in mortality.

**Table 7:** *k_t_* estimated for females (2019–2025) by the ARIMA model (0, 2, 3), with a confidence interval of 80% and 95%.

Year	Kt	Int Conf 80%		Int.Conf 95%	
		lim.inf	lim.sup	lim fin	lim.sup
2019	-5.697	-8.396	-2.998	-9.825	-1.570
2020	-6.508	-9256	-3.760	-10.711	-2.305
2021	-7.131	-9.901	-4.360	-11.367	-2.894
2022	-7.753	-10.549	-4.958	-12.029	-3.478
2023	-8.376	-11.201	-5.552	-12.696	-4.056
2024	-8.999	-11.856	-6.142	-13.369	-4.629
2025	-9.622	-12.516	-6.728	-14.048	-5.196

Data source: Ministry of Health of Cabo Verde.

**Table 8:** *k_t_* estimated for males (2019–2025) by the ARIMA model (0, 2, 2).

Year	Kt	Int Conf 80%		Int.Conf 95%	
		lim.inf	lim.sup	lim fin	lim.sup
2019	-7.55	-9.730	-5.380	-10.881	-4.229
2020	-8.380	-10.634	-6.125	-11.827	-4.932
2021	-9.204	-11.665	-6.744	-12.968	-5.441
2022	-10.029	-12.850	-7.209	-14.343	-5.716
2023	-10.854	-14.184	-7.524	-15.947	-5.761
2024	-11.679	-15.649	-7.709	-17.750	-5.608
2025	-12.504	-17.223	-7.784	-19.722	-5.286

Data source: Ministry of Health of Cabo Verde.

**Table 9:** *k_t_* estimated for both sexes (2019–2025) by the ARIMA model (2, 2, 3).

Year	Kt	Int Conf 80%		Int.Conf 95%	
		lim.inf	lim.sup	lim fin	lim.sup
2019	-3.940	-5.365	-2.514	-6.120	-1.760
2020	-7.057	-9.291	-4.824	-10.473	-3.642
2021	-5.578	-7.858	-3297	-9.065	-2.090
2022	-5.371	-7.943	-2.798	-9.305	-1.436
2023	-7.519	-10.646	-4.393	-1.2.301	-2.738
2024	-7.400	-10.713	-4.087	-12.466	-2.333
2025	-7.038	-10.586	-3.490	-12.464	-1.611

Data source: Ministry of Health of Cabo Verde.

Observed mortality is higher than projected over the study period.

## Discussion

Cape Verde showed a decreasing trend in the mortality rate, in the period from 1995 (4.3/1000) to 2017 (3.9/1000), with an increase in the year 2018 (4.5/1000). There was a positive evolution of standardized mortality from all causes from 2010 to 2018.

The Public health England, 2018 [17], described a downward trend in the number of deaths and mortality rates in the population with fluctuations in the number of deaths and rates recorded annually in its study that assessed trends over a period of 46 years and over a period of time. A South African study carried out by Pillay-van Wyk et al., 2016 over a 24-year period, demonstrated an increasing trend in the first nine (9) years of the analyzed period followed by a decline in the number of deaths which the authors associated with changes in HIV/AIDS mortality. Decreasing trends in mortality over time have been reported in other studies with variation in the periods and time of analysis [18–20].

Global statistics demonstrate significant gains in improved survival worldwide with increasing life expectancy and declining mortality rates resulting from improved health determinants [20,21]. Improved living conditions [22] and access to health services may explain the downward trend in mortality in Cabo Verde during the period under review. Furthermore, El Bcheraoui et al., 2020 [23] demonstrated that Cabo Verde had the best ranking in health in the Economic Community of West African States (ECOWAS) and one of the best in the African Continent in terms of quality indicators for health services.

The analysis of mortality distributed by sex revealed a higher incidence of deaths among men. The mortality rate for men is higher than for women, which corroborates the results of the study by Moura et al., 2015 [3]. The specific mortality rates by age group are high in individuals under the age of one year, with a decreasing trend in the 1 to 4-year age group and reach a minimum in the 5 to 9 age group, starting to increase in the 10 age groups. to 15 years and beyond. In the age group between 20 and 40 years, there was a marked increase in mortality in males, which corresponds to human mortality patterns for this age group [24,25].

Mortality rates in children under the age of five years show a decreasing trend over the years, in both sexes, which is in line with the results of Achoki et al., 2019, [26], who reported important gains in the reduction of rates infant mortality, a trend observed globally [27,28].

The mortality rate for women aged 50 to 54 years is similar to that for men aged 35 to 39 years, suggesting an earlier aging of men compared to women (15 years’ difference), which is in line with the results de Lenart et al., 2019, [16], although the differential in this study is 8 years (similarity between the rates of women aged 50 and men aged 42). This difference in mortality between the sexes was also observed in other studies [18,19,28–30]. According to Dicker et al., 2018, [28], the decrease in mortality rates in children and young people contributed to an overall decrease in mortality rates.

Over a 10-year period, Cabo Verde’s mortality rate decreased from 48.19 in 2010 to 46.88 per 10,000 inhabitants in 2018. The mortality trends of the six main groups of causes of death were analysed. The study showed that non-communicable diseases and trauma were responsible for more than 40% of deaths observed during the study period. The groups of diseases of the circulatory system accounted for about 25% of deaths, followed by tumours with 10.3% and 5.5% of deaths were due to trauma, poisoning and external causes [18]. They observed that cardiovascular diseases, tumours and diseases of the respiratory system were responsible for almost 70% of the proportion of deaths at the end of the study period and in their study, Ma et al., (2015), [19], the main specific causes of death that included heart disease, tumours, stroke, unintentional injuries, and diabetes.

The increase in morbidity and mortality from non-communicable diseases may be associated with the increase in the elderly population, evidenced by the increase in global life expectancy and other health determinants [21,31,32]. For example, in Cabo Verde, life expectancy increased from 48.6 to 72.9 years from 1960 to 2018 [33]. In developing countries, the burden of non-communicable diseases has increased but they do not yet predominate in the total disease burden [26].

The analysis revealed that cardiovascular diseases caused 37.1% of all deaths in women and 25.8% in men, had the highest mortality rate among the groups of causes considered during the entire period analysed. The standardized mortality rates in this group dropped from 13.3 to 13/10 thousand for women and from 18.4 to 17.1/10 thousand for men. Similar to the present study, Ma et al., (2015) and Coelho & Nunes, (2015) reported decreasing trends in mortality from cardiovascular diseases, respectively in the USA and Portugal, with men being more affected than women [18,19].

There was an increase in the mortality rate from tumours over time in Cabo Verde, with the highest rates among men and an almost static evolution among women, a trend similar to that reported by Coelho & Nunes, 2015 [18]. Tumours accounted for 10.3% of deaths observed over time. These account for 11.8% of deaths in Tanzania over a 10-year period, [34] contrary to the results of the current study, tumours were not among the top 10 causes of death in the South African study [29].

The increase may be explained by the increase in the elderly population, which is at greater risk of being diagnosed with the disease [32]. The differences in the ranking of tumours in the countries may be attributed to the different epidemiological profiles of the countries.

Injuries, traumas, poisonings and external causes had the greatest impact on male mortality rates, in 2005 the difference in rates between the sexes was 6.6 (8.1−1.5), currently it is 2.8 (3.5−0.7). Despite their decreasing trend in recent years, they continued to affect men more (18–20), which can be explained by the greater ease of men in engaging in dangerous and higher-risk activities, even in work situations [1,6]. External causes are among the 10 leading causes of death in French-speaking African countries in 2017 [23]. The analysis revealed that the mortality rate from injuries, injuries, poisoning and external causes was higher among males compared to females, but showed a decreasing trend (18–20).

Infectious and parasitic diseases accounted for 9.1% of deaths, respiratory diseases for 8.9% and, finally, unclassified clinical symptoms for about 20%.

Diseases of the respiratory system represent 8.9% of cases and occupy the 5th position in the group of causes of death. They show a growing trend for both sexes, as of 2005, and the male mortality rate is always higher than that of the female. Global statistics indicate an increase in the number of deaths from respiratory tract diseases but point to a decrease in mortality rates from respiratory tract diseases specifically Chronic Obstructive Pulmonary Diseases [35]. According with the study, smoking and particulate pollution were identified as the main mortality factors, especially in regions with a low sociodemographic index. Pollution and tobacco were ranked among the top 10 risk factors attributable to disability-adjusted life years in Kenya [26]. The increase in the burden of diseases of the respiratory system has been reported by several authors [18,19,30]. Studies in African countries have pointed lower respiratory infections and tuberculosis among the main causes of mortality [26,29,34].

In the present study, infectious and parasitic diseases accounted for 9.1 of the total deaths, higher in men, and with an increasing trend from 2005 to 2018 (increases from 4.8 in 2005 to 7.2 in 2018). The prevalence of infectious diseases as the main causes of mortality has been reported by several authors [23,26,29,34]. Other studies have reported rates of infectious and parasitic diseases in mortality [36,37].

In the current study, unclassified clinical symptoms accounted for 19.8% of the deaths observed, with a decreasing trend over time. There was a 70% decrease in the standardized mortality rate, from 13.7 to 4/10 thousand inhabitants from 2010 to 2018, which is in line with the trend reported by Coelho & Nunes, 2015b [18]. The downward trend could possibly be attributed to the improvement in the quality of registration, collection and processing of data on deaths in the country [38,39].

There were variations in the mortality profiles on each island, through the specific mortality rate by age group. It appears that mortality on the islands of São Nicolau, Boa Vista, Maio and Brava is higher when compared to the other islands, unlike the islands of São Vicente and Santiago, which have the lowest mortality rates. Geographical variations in mortality have also been reported by other studies [26,29,40].

The prevalence of non-communicable diseases over communicable diseases in Cape Verde, which shows that the country was in a phase of epidemiological transition [41].

Using the Lee-Carter model, it was found that 58.4% of the variation in the mortality rate is explained for females, 64% for males and 72.2% for both genders. However, this situation improves with the increase in the range of age groups (0–9, 10–19, 20–29, 30–39, …,90–89, 90+), obtaining a 74.2% variation explained in females, 61.9% in males and 70.6% in total (both sexes). It is noteworthy that these percentages are relatively low compared to 92% and 94% of the studies by Lee & Carter, Tuljapurkar, Li, & Boe, [12,42].

The kt index shows a trend of declining mortality over time. There is a greater sensitivity of mortality rates in relation to variations in kt in age groups below 25 years, and are greater in males when compared to females. A reduction in mortality from 2019 to 2025 is expected, with greater gains expected for males, aged 0 to 4 years, and a stagnation in mortality rates for the age group greater than or equal to 70 years in women, and for the age group greater than or equal to 60 years in men.

## Conclusions

There has been a very positive evolution in the mortality ratio in Cape Verde from 1995 to 2018. However, there was a disparity in the mortality rates between women and men. A high level of prematurity was found among men. There has been a very positive evolution with regard to the diagnosis of causes of death. Respiratory system diseases have increasing and the main cause of death in Cabo Verde has been diseases of the circulatory system, with a greater burden on women. Islands have different mortality profiles. São Nicolau, Brava and Santo Antão presented rates always above the national level, with circulatory diseases and unclassified clinical symptoms being the main causes of death. These islands have as a potential factor the difficulties in accessing health services, economic and financial difficulties and poor connection with the other islands.

A decrease in mortality rates is expected in the coming years for both sexes, being higher in males, especially in the younger age groups (0–4 years). Under existing conditions, gains are not expected in older age groups. These predictions should be considered with some caution, as this study has some weaknesses, such as the low percentage of mortality variation explained by the Lee-Carter prediction model.
